# Public support for policies to improve population and planetary health: A population-based online experiment assessing impact of communicating evidence of multiple versus single benefits

**DOI:** 10.1016/j.socscimed.2022.114726

**Published:** 2022-03

**Authors:** Eleni Mantzari, James P. Reynolds, Susan A. Jebb, Gareth J. Hollands, Mark A. Pilling, Theresa M. Marteau

**Affiliations:** a Behaviour and Health Research Unit, Department of Public Health and Primary Care, University of Cambridge, Cambridge, UK; b Nuffield Department of Primary Care Health Sciences, University of Oxford, UK

**Keywords:** Public support, Acceptability, Communication, Policy, Evidence, Benefit, Population health, Planetary health

## Abstract

**Background:**

Effective interventions for reducing the consumption of products that harm population and planetary health often lack public support, impeding implementation. Communicating evidence of policies’ effectiveness can increase public support but there is uncertainty about the most effective ways of communicating this evidence. Some policies have multiple benefits such as both improving health and the environment. This study assesses whether communicating evidence of multiple versus single benefits of a policy increases its support.

**Method:**

Participants (n = 4616) nationally representative of the British population were randomised to one of 24 groups in an online experiment with a 4 × 3 × 2 between-subjects factorial design. The messages that participants viewed differed according to the evidence they communicated (no message, effectiveness for changing behaviour, effectiveness for changing behaviour + one policy benefit, effectiveness for changing behaviour + three policy benefits), type of policy (taxation, availability) and the target behaviour (consumption of energy-dense food, alcohol, or meat). The primary outcome was policy support.

**Results:**

In a full factorial ANOVA, there was a significant main effect of communicating evidence of effectiveness on policy support, which was similar across policies and behaviours. Communicating three benefits increased support relative to communicating one benefit (d = 0.15; p = 0.01). Communicating one benefit increased support compared to providing evidence for changing behaviour alone (d = 0.13; p = 0.004) or no message (d = 0.11 p = 0.022).

**Conclusion:**

Communicating evidence of a policy's benefits increases support for policy action across different behaviours and policies. Presenting multiple benefits of policies enhances public support.

## Highlights

• Providing evidence of policies effectiveness at achieving policy goals increases public support.
• Uncertainty exists about the most effective ways of communicating this evidence.
• Evidence about multiple policy benefits increases support more than evidence for a single benefit.
• Effects are evident across behaviours and policies.

## 1 Introduction

Reducing consumption of energy-dense foods, meat and alcohol would improve health, including reducing rates of obesity and the risk of developing type II diabetes and many cancers (Afshin et al., 2019; Boada et al., 2016; Rehm et al., 2014; Salter, 2018; Stanaway et al., 2018; Stelmach-Mardas et al., 2016; Thomson et al., 2018; Traversy and Chaput, 2015; Y. C. Wang et al., 2011; World Health Organization, 2019; Yeomans, 2010). Achieving these health benefits would in turn lower the financial burden on health services associated with the costs of caring for these health conditions (Hall et al., 2015; Health, 2015; Laudicella et al., 2016; Mariotto et al., 2011; O'Connell and Manson, 2019; Park and Look, 2019; Stedman et al., 2020). Reducing consumption of these products would also lessen the environmental harms associated with their production, processing, transport and retail (Clark et al., 2019; Hallström et al., 2018; Mbow et al., 2019; Poore and Nemecek, 2018).

Excess consumption of energy-dense foods, meat and alcohol is often encouraged by structural factors, such as their low price and high availability (Cohen and Babey, 2012; Green et al., 2013; Jackson et al., 2010). Interventions that target these factors –such as taxation and reducing product availability– can reduce the consumption of these harmful products (Garnett et al., 2019; Hollands et al., 2019; Martineau et al., 2013; World Health Organization, 2009) but tend to receive less public support than information-based interventions (Petrescu et al., 2016; Sunstein et al., 2018), which are of limited effectiveness (Diepeveen et al., 2013). Public support has been defined as how individuals feel and think about the implementation or continued existence of a proposed government policy (e.g., Sekhon et al., 2017). Public support is one of several salient factors for policy makers considering implementing a policy, alongside the policy's effectiveness and likely costs (Cullerton et al., 2016; Freudenberg, 2014). Many theories and models attempt to capture the policy making process, including Advocacy Coalitions (Sabatier, 1988), Multiple Streams Framework (Kingdon and Stano, 1984), Punctuated Equilibrium (True et al., 2019) and the Narrative Policy Framework (Jones et al., 2014). These make different assumptions about the process-relevant actors, institutions, networks or subsystems, ideas or beliefs, policy context, and events, as well as the interactions between these factors that lead to policy stability or change over time, thus highlighting the complexity of the policy making process.

Although public support for policies known to be effective is often low, it appears amenable to change. Perceived effectiveness of a policy, defined as an individual's evaluation of the clarity, adequacy, and facilitation of the policy at achieving certain outcomes (Wan et al., 2014), is one of the strongest predictors of public support (Mazzocchi et al., 2015; Storvoll et al., 2015). Consistent with this, communicating evidence that a policy is effective at achieving a given goal increases support for policies across a range of domains, including population and planetary health (Reynolds et al., 2018, 2019). A meta-analysis found that the impact of communicating this information is equivalent to increasing public support from 50% to 54% (Reynolds et al., 2020). This effect appears reasonably robust, with little variation whether effectiveness information is presented alone or with other information, whether uncertainty is expressed, or whether effectiveness is asserted or described (quantitatively or qualitatively) (Reynolds et al., 2020).

Based on traditional social-cognitive models of behaviour, such as the theory of panned behaviour and the theory of reasoned action, attitudes reflect an individual's beliefs and values (Ajzen, 1991; Ajzen and Fishbein, 1988). Assuming policy support is a manifestation of an attitude towards a policy, increasing perceived policy effectiveness through communicating evidence of a policy's effectiveness, could in theory change attitudes towards a policy by changing policy-related beliefs and policy-related outcome expectancies. This is supported by the results of recent studies showing that perceived policy effectiveness has a significant and positive impact on policy attitudes (Chu et al., 2021; H. Wang et al., 2021). It should be noted, however, that the relationship between policy support and attitudes towards a policy is not necessarily straightforward. It is possible for example, for individuals to accept or tolerate policies they do not prefer, vote for those they consider bad or vote against those they consider good (PytlikZillig et al., 2018).

We have previously shown that communicating the effectiveness of a confectionery tax at reducing obesity levels can increase support for this policy (Reynolds et al., 2018). Additional analyses conducted on the publicly available dataset from this study showed that claiming that the tax was also effective at reducing health inequalities engendered further support. This suggests that one way of potentially increasing the impact of evidence of effectiveness information on policy support is by communicating the multiple benefits that arise from achieving a specific policy goal. To date, research has typically focused on the impact of communicating evidence of effectiveness at achieving a single benefit of interest, such as reducing obesity levels. No known studies have assessed the impact of communicating evidence of the multiple benefits a policy can have. This is important to elucidate, since most policies have many possible benefits, e.g. health-related, environmental, and financial, and most communication strategies, for example by media, governments, and corporations, present information on a range of these (Brick et al., 2018).

There are two possible mechanisms by which presenting multiple benefits might increase policy support. First, presenting information about multiple benefits might increase the likelihood that a benefit that is valued by the target audience is mentioned as achieved by the policy. This follows from evidence showing that different people value different outcomes as targets of government policies (Gollust et al., 2013) and that support for a policy is aligned with the value placed on the policy outcome (Franko et al., 2013; van der Linden et al., 2015). Along with beliefs, values are a core component of attitudes in traditional behaviour change theories, such as the theory of planned behaviours and the theory of reasoned action (Ajzen, 1991; Ajzen and Fishbein, 1988). A second possible mechanism is through repetition of the basic message that the policy is effective. This is consistent with findings from persuasion studies showing that a greater the number of arguments in a message increases message processing (Chaiken, 1980) and persuasiveness (Anderson, 1959; Willis, 1960) and that multiple similar messages increase positive attitudes toward the target of the message and message credibility (Cacioppo and Petty, 1989; Koch and Zerback, 2013; McCullough and Ostrom, 1974).

The aim of the current study was to assess the impact on public support of communicating evidence of a policy's effectiveness for achieving three compared with one benefit. It was hypothesised that support for policies is higher when one benefit is presented compared to no benefits and three benefits are presented compared to one benefit.

## 2 Methods

The study was preregistered with the Open Science Framework (https://osf.io/fa5x4/). Ethics approval was granted Cambridge Psychology Research Ethics Committee (Reference number PRE. 2020.020).

### 2.1 Design

An online study using a between-participants full factorial design with three factors resulting in 24 groups: Evidence of effectiveness (no message *vs* assertion of policy effectiveness *vs* assertion of policy effectiveness + one benefit *vs* assertion of policy effectiveness + three benefits) x Policy (availability *vs* taxation) x Behaviour (consumption of energy dense snacks *vs* consumption of meat *vs* consumption of alcohol). Participants were randomly allocated to one of the 24 groups using the research agency's software.

### 2.2 Participants

Participants (n = 4684) representative of the British population based on age, gender, socioeconomic status, region, education and recent voting behaviour, were recruited through a research agency (www.yougov.co.uk). Data were collected between the August 29 and September 3, 2020. Participants received £0.50 in compensation from the research agency for completing the study.

Sixty-eight participants (1.4% of the sample, balanced across groups) failed the attention check in which they were asked when they had last travelled to Mars (never, a few days ago, weeks ago and months ago; the correct answer being ‘never’) and were excluded from the analyses, giving a final sample of 4616 participants. The research agency provided sample weights that were used in all analyses to ensure the sample was representative of the British population. The weighted demographic characteristics of the sample are shown in Table 1. The number of participants randomised to each group (Table S1) and their demographic characteristics (Table S2) are presented in the supplemental material.

**Table 1:** Weighted demographic characteristics of sample (n = 4616).

**Age (years (sd))**	48.4 (17.0) (range 18–91; median = 49; IQR = 29)
**BMI (kg/m**^**2**^**(sd))**	27.1 (5.8) (range 13.9–35.3; median = 26.1; IQR = 6.92)
**Sex (n(%))**	
Female	2838 (51.6%)
Male	1778 (48.4%)
**Education**	
Low	1536 (33.3%)
Medium	867 (18.8%)
High	2014 (43.6%)
Prefer not to say	199 (4.3%)
**SES (n (%))**	
Low	1293 (28.0%)
Medium	2312 (50.1%)
High	1011 (21.9%)

**Education**

Low = no education, GCSEs or similar.

Medium = A-levels, non-degree teaching qualifications, or similar.

High = degree awards or higher.

**SES**

Low = DE.

Medium = C1C2.

High = AB.

**Income**

Low = up to £24,999.

Medium = between £25,000 and £49,999.

High = above £50,000.

From an earlier study (Reynolds et al., 2019), the mean expected effect size on policy support of a simple message that a policy is effective at changing the target behaviour compared to no message - the smallest predicted effect from the possible comparisons of the different levels of evidence communication- is d = 0.09. Based on this, a sample of 3768 (at least 157 participants in each of the 24 groups) provided 95% power at the 5%/13 significance level (using the Bonferroni adjustment - required given the multiple comparisons of interest) to detect a 3-way interaction term. The excess power allows for more than 13 comparisons. Power calculations were performed using R3.4.0. The final sample of 4616 exceeded this minimum sample.

### 2.3 Interventions

The interventions consisted of messages communicating evidence of multiple versus single policy benefits. Interventions were delivered online. There was no restriction imposed as to the type of device participants could use to access the study.

#### 2.3.1 Intervention description

Participants viewed one of 24 messages communicating evidence of effectiveness of policies for changing consumption-related behaviours. The messages differed according to the evidence that they communicated, the type of policy, and the targeted behaviour. Participants were allowed to read the messages for as long as they wished but were exposed to the message only once. On average, participants spent approximately 12 min reading the interventions and completing the questionnaire.

The four evidence communication messages were *i.* no message (control group); *ii*. a message asserting that the policy is effective at changing the target behaviour (control group); *iii.* a message asserting that the policy is effective at changing the target behaviour in addition to achieving one benefit (relating to population health; healthcare costs or planetary health, chosen at random); and *iv.* message asserting that the policy is effective at changing the target behaviour in addition to three benefits (relating to population health, healthcare costs and planetary health).

The two policies were: *i.* reducing the availability of the targeted products in supermarkets and increasing the availability of alternative products -low calorie snacks, plant-based foods and non-alcoholic drinks (Availability); *ii*. a tax to increase the price of the targeted product by 10% (Tax).

The three behaviours that were targeted were: *i* consumption of energy-dense snack foods, *ii.* consumption of meat, and *iii.* consumption of alcohol.

Examples of the messages presented to participants in each evidence of effectiveness communication group according to policy are presented in Box 1. (See the supplement for the complete set provided to the 24 groups).

Box 1 **Text presented to participants randomised to view messages about policies targeting the consumption of energy-dense snack foods**

**No message (control)**

***Tax***

*Imagine the government is considering a new policy to increase the price of high calorie snacks by 10% to help people eat less.*

***Availability***

*Imagine the government is considering a new policy to decrease the number of high calorie snacks and increase the number of low calorie snacks in supermarkets.*

**Evidence of effectiveness for changing target behaviour (control)**

***Tax***

*Imagine the government is considering a new policy to increase the price of high calorie snacks by 10% to help people eat less. Research shows that the introduction of this new policy will reduce the number of high calorie snacks people eat*.

***Availability***

*Imagine the government is considering a new policy to decrease the number of high calorie snacks and increase the number of low calorie snacks in supermarkets. Research shows that the introduction of this new policy will reduce the number of high calorie snacks people eat*.

**Evidence of effectiveness for changing target behaviour + one benefit**

***Tax***

*Imagine the government is considering a new policy to increase the price of high calorie snacks by 10% to help people eat less. Research shows that the introduction of this new policy will reduce the number of high calorie snacks people eat. This will cut (the number of people who get cancer or NHS costs or environmental harms).*

***Availability***

*Imagine the government is considering a new policy to decrease the number of high calorie snacks and increase the number of low calorie snacks in supermarkets. Research shows that the introduction of this new policy will reduce the number of high calorie snacks people eat. This will cut (the number of people who get cancer or NHS costs or environmental harms).*

**Evidence of effectiveness for changing target behaviour + three benefit**

***Tax***

*Imagine the government is considering a new policy to increase the price of high calorie snacks by 10% to help people eat less. Research shows that the introduction of this new policy will reduce the number of high calorie snacks people eat. This will cut the following:*

•*The number of people who get cancer.*
•*NHS costs*.
•*Environmental harms.*

***Availability***

*Imagine the government is considering a new policy to decrease the number of high calorie snacks and increase the number of low calorie snacks in supermarkets. Research shows that the introduction of this new policy will reduce the number of high calorie snacks people eat. This will cut the following:*

• *The number of people who get cancer.*
• *NHS costs*.
• *Environmental harms*.

Alt-text: Box 1

#### 2.3.2 Intervention development

The wording used to communicate evidence of a policy's effectiveness was based on that used in an earlier study (Reynolds et al., 2019). The benefits relating to each consumption behaviour were chosen from those cited in published government and research reports on the basis of them being common across the three behaviours. Through this process, it was identified that the target policies had benefits relating to three main domains: population health, healthcare costs and planetary health. The intervention was designed to include information about a benefit relating to each of these three domains. To test the prediction from the literature on persuasive communication that support increases with the number of benefits, with three claims considered optimal (Shu and Carlson, 2014), messages including information about a single benefit were also used. The wording used to describe these benefits was finalised following a pilot study and pre-pilot work (See supplemental material for aims and results of pre-pilot and pilot studies).

### 2.4 Measures

#### 2.4.1 Primary outcome

Support for the policy, assessed using one item rated on a 7-point scale, used in previous research (Reynolds et al., 2018):

“Do you support or oppose this policy”? (1 = Strongly oppose, to 7 = Strongly support)

#### 2.4.2 Secondary outcomes

1.Additional measure of support of the policy, assessed using one item rated on a 7-point scale, used in previous research (Reynolds et al., 2018):

“How much are you in favour of this policy being introduced? (1 = Strongly oppose, to 7 = Strongly favour). The secondary outcome was the mean of the two measures of support (primary and secondary; Cronbach's alpha = 0.97).

2.Perceived effectiveness of the policy at changing the target behaviour, using two items (Cronbach's alpha = 0.92) rated on a 7-point scale (1 = Strongly disagree, to 7 = Strongly agree) used in previous research (Reynolds et al., 2018):

“The new policy will reduce [behaviour]”

“The new policy will help solve the UK's problem with [behaviour]”

#### 2.4.3 Demographic characteristics and other variables

1.Value assigned to different benefits (population health vs healthcare costs vs planetary health), assessed using a ranking measure and a rating measure. The ranking measure asked participants to choose the most important and second most important benefit to them. The rating measure asked participants to rate on a 7-point scale how important each benefit was to them.

Details on the exact measures are available in the supplement.

2. ;Weekly consumption of the target products (seldom or never; less than once a week 1–2 times a week; 3–4 times a week; 5 or more times a week) assessed using five items assessing high-energy snacks, two items assessing meat products and four items assessing alcoholic drinks. Items measuring each product type (snack consumption: Cronbach's alpha = 0.71; meat consumption: Cronbach's alpha = 0.55; alcohol consumption: Cronbach's alpha = 0.44) were combined by multiplication to get variables relating to overall snack consumption, overall meat consumption and overall alcohol consumption. Each variable was then divided unto tertiaries representing low, moderate and heavy consumption.
3. Demographic characteristics: age, education level, income level, and self-reported weight and height, to estimate BMI. Educational achievement was recoded into three categories: low education (no education, GCSEs or similar); medium education (A-levels, non-degree teaching qualifications, or similar); and, high education (degree awards or higher). Socio-economic status was also recoded into three categories: low (DE), medium (C1C2), and high (AB).

### 2.5 Data analysis

The full statistical analysis plan was pre-registered with the OSF (https://osf.io/gvwhx/).

The demographic characteristics of participants, as well as primary and secondary outcomes were described [means (SDs); proportions (95%CI)] (Table 1, Table 2; Supplemental material Tables S2-S4).

**Table 2:** Policy support [% (95% confidence intervals)] for each policy by targeted behaviour according to level of evidence of effectiveness communication.

**Evidence of effectiveness**												
	No message			Effectiveness for changing behaviour			Effectiveness for changing behaviour + one benefit			Effectiveness for changing behaviour + three benefits		
**Behaviour**	Tax	Availability	Across policies	Tax	Availability	Across policies	Tax	Availability	Across policies	Tax	Availability	Across policies
Energy-dense food consumption	37% *(31–44%)*	59% *(52%-66%)*	48% *(43%-53%)*	36% (*30%-43%)*	55% *(47%-62%)*	45% *(40%-50%)*	48% *(40%-55%)*	63% *(56%-70%)*	55% *(51%-60%)*	54% *(47%-61%)*	60% (*53%-67%)*	57% *(52%-62)*
Meat consumption	18% *(13%-24%)*	36% *(29%-43%)*	27% *(23%-32%)*	24% *(18%-30%)*	28% *(21%-35%)*	26% *(21%-30%)*	24% *(17%-30%)*	35% *(28%-41%)*	29% *(25%-34%)*	36% *(29%-41%)*	43% *(36%-50%)*	40% *(35%-45%)*
Alcohol consumption	41% *(34%-48%)*	32% *(26%-39%)*	36.5% *(32%-41%)*	37% *(30%-44%)*	30% *(24%-37%)*	33% *(29%-38%)*	41% *(34%-48%)*	34% *(27%-40%)*	37% *(33%-42%)*	44% *(37%-50%)*	53% *(46%-60%)*	48% *(43%-53%)*
Across behaviours	32% *(28%-36%)*	42% *(38%-46%)*	37% *(34%-40%)*	32% *(28%-36%)*	38% *(34%-42%)*	35% *(32%-38%)*	38% *(34%-42%)*	44% *(40%-48%)*	41% *(38%-44%)*	45% *(41%-49%)*	52% *(48%-56%)*	48% *(45%-51%)*

The primary analysis tested the hypothesis that public support for a policy increases with the number of benefits for which evidence of effectiveness is presented, using a full factorial 4 × 3 × 2 ANOVA. This assessed the main effect of different levels of evidence communication on policy support, across behaviours -i.e. pooling together data relating to messages describing policies to tackle excess consumption of energy-dense snacks, meat and alcohol, as well as across policies –i.e. pooling together data relating to messages describing the two different policies of taxation and availability. Interaction terms between policy, behaviour and evidence communication were added to the model to explore whether any effects of asserting evidence of effectiveness of a policy for achieving one benefit vs three benefits differ according to policies and behaviours. Bonferroni adjusted pairwise comparisons were used to indicate if support significantly differed across the levels of each factor. Model diagnostics were acceptable (normality of distributions of residuals confirmed through inspection of histogram and QQ plots, available in the supplement). There were no outliers (±3SDs from the mean) in the data and no missing data relating to the primary outcome.

To ensure the reliability of the results, two sets of sensitivity analyses were conducted i) using a composite measure of support, consisting of the mean of the two items as the dependent variable; ii) controlling for demographic variables and frequency of consumption of the targeted products.

To aid interpretation of the results, data relating to policy support were dichotomised (1–4 = 0, 4.01–7 = 1) to indicate the proportions of participants who found the policy acceptable (i.e. those rating above the scale midpoint).

The above analyses were repeated with perceived policy effectiveness as the dependent variable, to assess the main effect of different levels of evidence communication on this secondary outcome. Model diagnostics were acceptable (see supplement). There were no outliers in the data and no missing data relating to the secondary outcome.

Exploratory moderation analyses were conducted to assess whether any impact of asserting evidence of policy's effectiveness at achieving one benefit, compared to the ‘no message’ group, on policy support was moderated by the value assigned to that benefit. Data were pooled for messages relating to the three different behaviours and the two different policies. Analyses involved six regressions using the PROCESS macro (Hayes, 2017) in SPSS. The models included policy support as the dependent variable and the interaction between evidence and perceived importance (using two measures: a ranking and a rating measure, assessed separately as a potential moderator) assigned to achieving each benefit (population health, planetary health, and healthcare). Diagnostics of all six models were acceptable.

## 3 Results

The randomisation resulted in balanced groups in terms of number of participants allocated to each and demographic variables (Tables S1 and S2 of the supplement).

### 3.1 Policy support

The proportion of participants in each group who supported the policy is presented in Table 2. Mean policy support scores according to each group are presented in the supplement (Table S3).

Policy support varied significantly with the evidence of effectiveness communicated (F (3, 5185) = 22.3, p < 0.001), the policy type (F (1, 5185) = 56.8, p < 0.001) and the behaviours targeted (F (2, 5185) = 107.6, p < 0.001) (Table 3).

**Table 3:** ANOVA results: Policy support according to evidence of effectiveness communication, policy type and target behaviour.

**Predictor**	**Sum of Squares**	**df**	**Mean Square**	**F**	**P**	**Partial Eta Squared**	**Cohen's f**
Intercept	88910.059	1	88910.059	27914.503	0.000	0.843	
Evidence of effectiveness communication	212.818	3	70.939	22.272	0.000	0.013	0.013
Policy type	181.015	1	181.015	56.832	0.000	0.011	0.011
Behaviour targeted	685.724	2	342.862	107.646	0.000	0.040	0.040
Evidence of effectiveness communication * Policy type	5.454	3	1.818	0.571	0.634	0.000	0.000
Evidence of effectiveness communication * Behaviour targeted	8.968	6	1.495	0.469	0.832	0.001	0.001
Policy type * Behaviour targeted	106.848	2	53.424	16.773	0.000	0.006	0.006
Evidence of effectiveness communication * Policy type * Behaviour targeted	29.593	6	4.932	1.549	0.158	0.002	0.002
Error	16514.665	5185	3.185				
R Squared = .069 (Adjusted R Squared = .065)							

Policy support was greater when communicating evidence that a policy was effective at achieving three benefits compared to one (mean difference: -0.27, 95%CI -0.54 to -0.09; d = 0.15; p = 0.011). Communicating evidence that a policy was effective at achieving one benefit increased support compared to evidence of effectiveness for changing behaviour alone (mean difference; -0.24, 95%CI -0.43 to -0.06; d = 0.13; p = 0.004) or not providing any effectiveness message (mean difference: -0.20, 95%CI -0.39 to -0.02; d = 0.11; p = 0.022).

There were no significant interactions (3-way or 2-way) between communicating evidence of a policy's effectiveness, the type of policy or the behaviour targeted. There was a significant interaction between policy type and the behaviour targeted (Table 3).

**Table 4:** Proportion of participants [95%CI (n)] selecting each benefit as most important, second most important and third most important.

**Benefit**			
**Ranking**	Population Health	Planetary Health	Healthcare Costs
Most important	51% [49%–53% (2377)]	23% [20%–25% (1051)]	26% [23%–28% (1188)]
Second most important	31% [28%–33% (1426)]	42% [40%–44% (1921)]	27% [24%–29% (1269)]
Third most important	18% [15%–21% (814)]	36% [34%–28% (1643)]	47% [45%–49% (2159)]

In pre-planned sensitivity analyses, the direction of the results was unchanged when using a composite measure of policy support, consisting of the mean of the two items (alpha = 0.97) and after adjusting for demographic variables (age, education, ethnicity and income) and frequency of consumption of targeted products (energy-dense snacks, meat and alcohol) (Supplemental material S3).

### 3.2 Perceived policy effectiveness

Perceived policy effectiveness scores according to each group are shown in the supplement (Table S4).

Perceived policy effectiveness varied significantly with the evidence of effectiveness communicated (F (3, 5185) = 9.1, p < 0.001), the policy type (F (1, 5185) = 63.8), p < 0.001 and the targeted behaviour (F (2, 5185) = 30.1, p < 0.001) (supplement Table S5).

Perceived policy effectiveness was higher when communicating evidence that a policy was effective at achieving one benefit (mean difference: -0.21, 95%CI -0.37 to -0.05; p = 0.003) or three benefits (mean difference −0.31, 95%CI -0.46, -0.15; p < 0.001) compared to not providing any effectiveness message. There was no significant difference of communicating evidence that a policy was effective at achieving one benefit compared to three benefits (mean difference = −0.96, 95%CI -0.25 to 0.06; p = 0.639). There were no significant interactions (3-way or 2-way) between communicating evidence of a policy's effectiveness, the type of policy or the behaviour targeted (Table S5).

### 3.3 Exploratory moderation analysis

The percentage of participants selecting each benefit (population health, planetary health, reducing healthcare costs) as i) the most important; ii) second most important and iii) third most important are presented in Table 4. The mean importance scores for each benefit are in the supplement (Table S6).

There was no evidence to suggest that the impact on policy support of communicating evidence of policy's effectiveness for achieving one benefit, compared to not providing any message, was moderated by the value assigned to that benefit. This was true regardless of which benefit was included in the analyses (population health, planetary health or healthcare costs) or which measure of importance was included as the potential moderator (ranking or rating measure) (Supplemental material).

## 4 Discussion

Public support for policies intended to reduce consumption of energy dense food, alcohol or meat is increased by communicating evidence first that they are effective at achieving that goal, and second that they have benefits for population and planetary health. Support is higher when evidence about multiple benefits is communicated compared to a single benefit. Communicating evidence of a policy's benefits also increases perceptions that the policy is effective. These effects on policy support and perceived policy effectiveness appear generalizable across policies and behaviours.

These results confirm and extend previous studies showing the impact on public support of communicating evidence of a policy's effectiveness targeting population and planetary health (Reynolds et al., 2020; (Reynolds et al., 2019; Reynolds et al., 2018; Reynolds et al., 2020). The meta-analysis by Reynolds et al. (2020) estimates the effect size of this intervention as four percentage points, for example increasing support from 50% to 54%. The current study replicates both the effect and the effect size, with support increasing by four percentage points when communicating evidence of a single benefit (from 37% to 41%). Most importantly, the current study showed that support can be meaningfully increased by almost two-fold when information about three policy benefits is communicated compared to only one (from 41% to 48%) and by 11 percentage points compared to when no benefits are presented (from 37% to 48%). The fact that these effects are generalizable across policy types and target behaviour suggests that this intervention could be used to increase support for a range of policies. The ideal number of benefits and whether or not support could be further increased with presentation of additional policy benefits remain empirical questions.

There are two potential mechanisms to account for the increase in support from communicating multiple benefits. First, it increases the likelihood that a benefit valued by an individual will be described. This in turn, increases the likelihood that an individual will support the policy, as it targets a problem salient to them. However, we found no evidence here that the impact on policy support was moderated by the value assigned to that benefit, these analyses were purely exploratory and the null findings could be the result of insufficient power. Second, presenting multiple benefits might increase public support through repetition of the basic message that the policy is effective. This is based on findings from persuasion studies showing that a greater number of arguments included in a message increases belief and attitude change (Anderson, 1959; Cacioppo and Petty, 1989; Chaiken, 1980; Koch and Zerback, 2013; McCullough and Ostrom, 1974; Willis, 1960). But we found no evidence of a difference in perceptions of effectiveness between those presented with information about multiple policy benefits compared to those presented with information of a single benefit. Further research is needed to elucidate the potential mechanisms by which presentation of information about multiple benefits increases policy support.

The current study is the first known to assess the impact of communicating evidence of multiple compared to single policy benefits on policy support. The increase in support demonstrated here might seem relatively small, but when applied at a population level it may have large and important consequences (Prentice and Miller, 1992). To put this into context, consider, for example, public support for the COVID-19 vaccine, uptake of which is important for managing the current pandemic. An 11% increase in support, found in the current study when presenting information about multiple benefits, could contribute to wider efforts at encouraging vaccine uptake, bringing coverage closer to the levels needed to achieve herd immunity against the disease. While the exact level of public support required to influence policy makers to implement a policy is unknown, the results of the present study reinforce previous findings that communicating evidence of policy effectiveness can increase support for a range of policies. Most effective interventions tend to be the least supported (Diepeveen et al., 2013). Communicating policy effectiveness could align public support more closely with a policy's effectiveness. Communicating evidence of a policy's effectiveness is also likely to increase policy-makers’ support for implementing policies (Ashcraft et al., 2020; Cairney and Kwiatkowski, 2018; Purtle et al., 2019).

The findings of the current study can be readily applied to communication strategies intended to increase support for any policy. Policy advocates – from public health advocacy groups, non-governmental organisations, and those working within governments – could develop their messaging to include multiple benefits of the policy being advocated. The current study provides novel evidence that this should increase support, over and beyond simply sharing evidence that it is effective (Reynolds et al., 2020).

The strength of the current study lies in its large full factorial between-subjects design, which ensured ratings of support were not influenced by prior exposure to other policies or target behaviours, and its large sample size. Detailed research was conducted prior to running the study to identify the policy benefits to be used -- chosen from those cited in published government and research reports on the basis of them being common across the targeted behaviours-- as well as the wording to describe these benefits, -- based on extensive piloting work. Although effects generalised to two different polices within three different health domains (population health, planetary health, healthcare quality), future research could expand this approach and assess the impact on support for policies in non-health related fields. Furthermore, although the sample was representative of the British population in terms of demographics, it derived from the online panel of a research agency, implying a potential self-selection bias, which might have hindered the sample's representativeness in terms of beliefs and values.

In conclusion, communicating evidence of a policy's benefits arising from the reduction in consumption of energy dense foods, meat, and alcohol, increases public support for that policy. Support is highest when evidence about multiple benefits is presented. Communicating evidence of a policy's benefits also increases perceptions of policy effectiveness. The effects on public support for a policy and perceived policy effectiveness appear generalizable across behaviours and policies. These results add to the existing evidence for communicating evidence of effectiveness of policies, suggesting that this is a reliable intervention to increase public support for a range of policies.

## Author statement

**Eleni Mantzari**: Conceptualization, Methodology, Investigation, Validation, Formal analysis, Data curation, Writing – original draft. **James P Reynolds**: Conceptualization, Methodology, Writing – review & editing. **Susan A Jebb**: Conceptualization, Methodology, Writing – review & editing.**Gareth J Hollands**: Conceptualization, Methodology, Writing – review & editing. **Mark A Pilling**: Methodology, Formal analysis, Writing – review & editing. **Theresa M Marteau**: Conceptualization, Methodology, Writing – review & editing, Supervision

## Ethics approval

Approved by the Psychology Research Ethics Committee of the University of Cambridge (Reference Number: PRE. 2020.020).

## Availability of data and material

The datasets generated and analysed during the current study are available on the Open Science Framework project page (link to be added once accepted).

## Funding

This research was funded in whole, or in part, by the 10.13039/100010269Wellcome Trust [ref: 206853/Z/17/Z].

For the purpose of Open Access, the author has applied a CC BY public copyright licence to any Author Accepted Manuscript version arising from this submission.

## Declaration of competing interest

The authors declare that they have no competing interests.
