# Supplementary material for: Public support for policies to improve population and planetary health: A population-based online experiment assessing impact of communicating evidence of multiple versus single benefits
Source: Soc Sci Med. 2022 Mar;296:114726. doi: 10.1016/j.socscimed.2022.114726 (PMC8907862; doi:10.1016/j.socscimed.2022.114726)
Supplement: Multimedia component 1 [file mmc1.docx]

**Public support for policies to improve population and planetary health: impact of communicating evidence of multiple versus single policy benefits**

**Supplemental Material**

**Pre-pilot study to develop messages for pilot study**

**Aims**

To assess the impact on message believability and coherence of the following:

1. explanations for how the target behaviour causes adverse outcomes that do and do not include an additional statement that changing the target behaviour has benefits *i.e.* reverses the adverse outcomes.
2. explanations that include or avoid wording that can be interpreted as attributing individual responsibility for the target behaviours.
3. for messages referring to planetary health, explanations that do and do not include a statement linking carbon emissions to planetary health

**Methods**

Design

Mixed between and within-subjects.

*Within-subjects factor:*

Benefits - 3 levels:

- reduced cancer rates
- reduced healthcare costs
- reduced carbon emissions

*Between-subjects factor:*

Message type^[[1]](#footnote-1)^ - – 4/7* levels:

- message that does not include additional statement about the reversal of adverse outcomes (Non-Reversal)
- message that includes additional statement about reversal of adverse outcomes (Reversal)
- message that attributes individual responsibility for behaviour (Attribution)
- message that does not attribute individual responsibility for behaviour (Non-attribution)

*three additional levels for benefits relating to planetary health^[[2]](#footnote-2)^:

- message that includes mention of carbon emissions only
- message that includes mention of environmental harms
- message that includes an explicit explanation of the link between carbon emissions and environmental harms

Participants

291 recruited through Prolific Academic. This sample size allowed us to detect an effect size (Cohen’s d) of 0.27 for the interaction between the wording of messages and the different benefits, using an ANCOVA (alpha 5%, power 80%) for each measure.

Messages

These are shown in Box A below.

Measures

1. Believability (primary outcome)

This was assessed by rating this statement “*The messages related to eating and drinking and cancer/NHS costs, carbon emission were…”*on three, 7 point scales, anchored at either end by:

- - 1. believable/unbelievable,
    2. convincing/unconvincing
    3. trustworthy/untrustworthy:

This was adapted from similar measures used in previous studies (Beltramini, 1988; Berry et al., 2018; O'Cass & Griffin, 2006).

- 1. level of coherence

1. Perceived coherence comprising three items rated on a 7-point scale, adapted from previous research (Bishop et al., 2005):

*“To what extent do you understand how eating and drinking less can cut the number of people who get can cancers/NHS costs/ carbon emissions?”*

1 = do not understand at all; 7 = completely understand

*“How easy would you find it to explain to someone else exactly how eating and drinking less can cut the number of people who get can cancers/NHS costs/carbon emissions?*

1 = not at all easy; 7 = extremely easy

*To what extent do you have a clear picture of how s can cut the number of people who get can cancers/NHS costs/carbon emissions?*

1. = not at all; 7 = completely clear picture
2. Importance of cutting carbon emissions vs environment harms

This was assessed by asking participants to rate on a 7-point scale how important each is: *“How important is achieving cutting carbon emissions/environment harms to you*?” 1= very unimportant; 7=very important

Procedure

Participants viewed information relating to one behaviour (consumption of energy-dense snacks) and one policy (taxation). See Box A for messages that were pre-piloted. Each participant was asked to read three messages, one relating to each policy benefit. The message type was determined by randomisation: for benefits relating to population health and healthcare costs, participants were randomised to one of four message types; for planetary health, they were randomised to one of seven different message types. Each message was rated on believability and coherence. After completing the ratings participants were asked to rate the importance of cutting each environmental harms vs carbon emissions.

*Analysis*

The messages that were selected for the pilot study were those with the highest scores on the primary outcome measure – believability with consideration also given to scores on coherence. This was examined by computing both statistical significance (i.e. *p* < .05) and Cohen’s *d,* which had to be at least *d* = .05 to conclude that there were meaningful differences between groups *i.e.* types of messages.

**Results**

**Impact on message believability and coherence of message wording** (Non-Reversal vs Reversal vs Non-attribution)

Means (sd) believability and coherence scores according to wording condition overall and for each benefit

|  | **Wording condition** | | |
| --- | --- | --- | --- |
|  | Non-reversal (n=97) | Reversal (n=99) | Non-attribution (n=95) |
| **Believability**  Overall*  Population health  NHS costs  Planetary health | 4.43 (1.21)  4.25 (1.57)  4.72 (1.50)  4.32 (1.62) | 4.46 (1.17)  4.25 (1.63)  4.84 (1.45)  4.27 (1.77) | 4.30 (1.18)  4.02 (1.65)  4.83 (1.39)  4.05 (1.68) |
| **Coherence**  Overall*  Population health  NHS costs  Planetary health | 4.55 (1.18)  4.12 (1.74)  5.26 (1.45)  4.27 (1.72) | 4.56 (1.26)  3.99 (1.76)  5.27 (1.51)  4.42 (1.89) | 4.51 (1.23)  3.96 (1.94)  5.23 (1.55)  4.32 (1.75) |

*Across benefits

**Believability**

Mixed methods ANOVA: No main effect of wording condition F(2, 288)= 0.464, p>0.05.
No interaction between wording condition and benefit F(4, 586)= 1.089, p>0.05.

**Coherence**

Mixed methods ANOVA: No main effect of wording condition F(2, 288)= 0.464, p>0.05
No interaction between wording condition and benefit F(4, 586)= 1.089, p>0.05

**Impact on message believability and coherence of using different terms to describe benefits relating to Planetary Health** (Carbon Emission vs Environmental Harms vs Both)

Means (sd) believability and coherence scores according to different planetary health terms different terms

|  | **Planetary health message** | | |
| --- | --- | --- | --- |
|  | Carbon emissions (n=162) | Environmental harms (n=67) | Both (n=63) |
| **Believability** | 4.17 (1.63) | 4.27 (1.88) | 4.21 (1.60) |
| **Coherence** | 4.16 (1.75) | 4.49 (1.93) | 4.58 (1.61) |

No significant impact of different terms to describe on believability F(2, 288)=0.225, p>0.05 or coherence F(2, 288)=1.519, p>0.05

Reducing Environmental hams was rated as more important than reducing carbon emissions (Environmental harms mean: 6.16 (sd=0.95); Carbon emissions: 5.99 (sd=1.05), t-paired (289) = -3.719, p<0.001))

**Conclusions**

There were no meaningful differences in overall believability and coherence scores between the non-reversal and reversal groups. The non-attribution statements resulted in a decrease in scores. Statements including mention of ‘environmental harm’ increased message believability and coherence and this outcome was rated as more important than cutting carbon emissions.

Based on these results, for the pilot study it was decided to use messages that do not include an additional statement about the reversal of adverse outcomes (Non-Reversal) and for messages relating to planetary health it was decided to use the term ‘environmental harms’.

**Box 1 Messages assessed in Pre-Pilot study**

| **Message describing evidence of effectiveness at achieving target behaviour:**  *The government is considering a new policy to increase the price of energy-dense snacks, such as confectionary and chocolate, by 10% to help people eat less. Research shows that the introduction of this new policy will reduce the number energy-dense snacks people eat.*  **Options to describe benefits:**  ***Health***   1. *This will cut the number of people who get cancer caused by eating too much.*   *OR*   1. *This will cut the number of people who get cancer caused by eating too much. If people ate and drank less, fewer people would have health problems.*   *OR*   1. *This will cut the number of people who get caused by eating too much. The policy would help people eat less, meaning fewer people would have health problems).*   ***Healthcare costs***   1. *This will cut NHS costs caused by eating too much.*   *OR*   1. *This will cut NHS costs caused by eating too much. If people ate less, there would be fewer health problems needing treatment.*   *OR*   1. *This will cut NHS costs caused by eating too much. The policy would help people eat less, meaning there would be fewer health problems needing treatment.*   ***Planetary health***   1. *This will cut carbon emissions caused by producing, processing and transporting food.*   *OR*   1. *This will cut carbon emissions caused by producing, processing and transporting food. If people ate less there would be fewer carbon emissions.*   *OR*   1. *This will cut carbon emissions caused by producing, processing and transporting food. If people ate less, there would be fewer harms to the environment.*   *OR*   1. *This will cut carbon emissions caused by producing, processing and transporting food. If people ate less, there would be fewer carbon emissions and therefore fewer harms to the environment.*   *OR*   1. *This will cut carbon emissions caused by producing, processing and transporting food. The policy would help people eat less, meaning there would be fewer carbon emissions.*   *OR*   1. *This will cut carbon emissions caused by producing, processing and transporting food. The policy would help people eat less, meaning there would be fewer harms to the environment.*   *OR*   1. *This will cut carbon emissions caused by producing, processing and transporting food. The policy would help people eat drink less, meaning there would be fewer carbon emissions and therefore fewer harms to the environment.* |
| --- |

**Pilot Study to Develop Messages**

**Aim**

To resolve uncertainty around the optimal wording of messages to describe each of three benefits. Specifically, to assess the impact of using short messages compared to longer messages that make explicit the causal link between the target behaviour and the benefits on:

- Perceived policy effectiveness
- Importance of achieving target outcome
- Policy support
- Perceived coherence

**Methods**

Design

The study had a between and within subjects design:

*Between-subjects factor:* wording of messages (2 levels: short; detailed) x benefits (4 levels: reduced cancer rates; reduced healthcare costs; reduced carbon emissions; all three combined) – 8 groups

*Within-subjects factor:* behaviour (3 levels: consumption of energy-dense snacks; meat; alcohol)

Participants

1000 participants were recruited from Prolific Academic, an online platform. This sample size allowed us to detect an effect size (Cohen’s d) of up to 0.105 for the interaction between the wording of messages and the different benefits, using an ANOVA (alpha 5%, power 80%) for each between-subjects measure.

Messages

See Box 2

Measures

Primary outcome

1. Perceived policy effectiveness (primary outcome) rated on a 7-point scale:

“*How effective do you think this policy is in reducing [target outcome]?*

1= very ineffective; 7=very effective

Secondary outcomes

1. Importance rated on a 7-point scale:

*“How important is achieving this outcome to you*?”

1= very unimportant; 7=very important

1. Policy support rated on a 7-point scale:

*“How much are you in favour of this policy being introduced?”*

1 = strongly oppose; 7 = strongly favour

1. Perceived coherence comprising three items rated on a 7-point scale, adapted from previous research (Bishop, Marteau, Hall, Kitchener, & Hajek, 2005):

*“To what extent do you understand how eating and drinking less can cut the number of people who get can cancers/NHS costs/ carbon emissions?”*

1 = do not understand at all; 7 = completely understand

*“How easy would you find it to explain to someone else exactly how eating and drinking less can cut the number of people who get can cancers/NHS costs/carbon emissions?*

1 = not at all easy; 7 = extremely easy

*To what extent do you have a clear picture of how s can cut the number of people who get can cancers/NHS costs/carbon emissions?*

1 = not at all; 7 = completely clear picture

Procedure

Each participant read and rated three separate messages, one relating to each target behaviour. The content of the messages and the order in which they are presented was determined by randomisation.

Analysis

The messages selected for the main study were those with the highest scores on the primary outcome measure – perceived policy effectiveness - with consideration also given to scores on the other outcome measures – Coherence, Importance and Policy support. This was examined by computing both statistical significance (i.e. *p* < .05) and Cohen’s *d,* which had to be at least *d* = .05 to conclude that are meaning differences between groups *i.e.* types of messages.

The intention wasto use either short or longer messages in the main study to describe each of the three policy domains - excess consumption of energy-dense snack foods, meat and alcohol.

**Results**

Below is a description of the main results. Detailed results are given fort the primary outcome – perceived policy effectiveness.

=

- Overall (i.e. across benefits), shorter explanations resulted in meaningful (but not significant) increases in perceived policy effectiveness (primary outcome)
- Overall, shorter explanations resulted in meaningful (but not significant) increases in policy support and message believability (secondary outcomes)
- Overall, longer explanations resulted in significant increases in message coherence (secondary outcome).

**Primary outcome: Perceived effectiveness of policy**

Mean (sd) perceived effectiveness scores for short and long explanations according to each benefit and overall (across benefits)

| **Benefit** | **Wording** | **Mean** | **Std. Deviation** | **N** | **d effect size** |
| --- | --- | --- | --- | --- | --- |
| Population Health | Short | 4.3575 | 1.52674 | 124 | 0.02 |
|  | Long | 4.3937 | 1.49273 | 127 |  |
| NHS costs | Short | 4.4436 | 1.20921 | 127 | 0.15* |
|  | Long | 4.2336 | 1.52308 | 127 |  |
| Planetary Health | Short | 4.4844 | 1.34370 | 128 | 0.09* |
|  | Long | 4.3600 | 1.40633 | 125 |  |
| Combined | Short | 4.4541 | 1.41961 | 127 | 0.01 |
|  | Long | 4.4630 | 1.43665 | 126 |  |
| Overall | Short | 4.4354 | 1.37537 | 506 | 0.05* |
|  | Long | 4.3624 | 1.46371 | 505 |  |

*Meaningful comparison i.e. d ≥ 0.05)

No main effect of wording condition (Short vs Long) F(1, 1003)= 0.653, p>0.05. No interaction between wording and benefit F (3, 1003)=0.417, p>0.05.

Shorter explanations resulted in a meaningful increase in perceived effectiveness scores overall and for messages relating to NHS costs and Planetary Health.

**Conclusion**

Based on the results it was decided to use short message to describe policy benefits in the main study

**Box 1 Messages assessed in Pilot study**

| **Message describing evidence of effectiveness at achieving target behaviour:**  *The government is considering a new policy to [increase the price of alcoholic drinks by 10%/reduce the number of alcoholic drinks and increase the number of alcohol free drinks in supermarket]) to help people drink less. Research shows that the introduction of this new policy will reduce the number of people who drink.*  **Options to describe benefits:**  ***Health***  *Short*  *This will cut the number of people who get cancer*  *Long*  *This will cut the number of people who get cancer caused by eating and drinking too much.*  ***Healthcare costs***  *Short*  *This will cut NHS costs*  *Long*  *This will cut NHS costs caused by eating and drinking too much.*  ***Planetary health***  *Short*  *This will cut environmental harms*  *Long*  *This will cut environmental harms*  *caused by producing, processing and transporting food and drink.*    Combined benefits^[[3]](#footnote-3)^  *Short*  *This will cut the following:*   - *the number of people who get cancer* - *NHS costs* - *Environmental harms*   *Long*  *This will cut the following:*   - *The number of people who get cancer, caused by eating and drinking too much* - *NHS costs.*    - *Caused by eating and drinking too much*   - *If people ate and drank less, there would be fewer health problems needing treatment.* - *Environmental harms.*    - *caused by producing, processing and transporting food and drink.*   - *If people ate and drank less, there would be fewer carbon emissions.* |
| --- |

**Intervention messages according to experimental group**

**CONTROL**

**Group 1a: No message control – consumption of energy-dense snacks – tax**

*Imagine the government is considering a new policy to increase the price of high calorie snacks by 10% to help people eat less.*

**Group 1b: No message control – consumption of meat – tax**

*Imagine the government is considering a new policy to increase the price of meat products by 10% to help people eat less.*

**Group 1c: No message control – consumption of alcohol – tax**

*Imagine the government is considering a new policy to increase the price of alcoholic drinks by 10% to help people drink less.*

**Group 2a: No message control – consumption of energy-dense snacks – availability**

*Imagine the government is considering a new policy to decrease the number of high calorie snacks and increase the number of low calorie snacks in supermarkets.*

**Group 2b: No message control – consumption of meat – availability**

*Imagine the government is considering a new policy to decrease the number of meat products and increase the number of plant-based foods in supermarkets.*

**Group 2c: No message control – consumption of meat – availability**

*Imagine the government is considering a new policy to decrease the number of alcoholic drinks and increase the number of alcohol-free drinks in supermarkets.*

**BEHAVIOUR CHANGE**

**Group 3a: Achieving behaviour change – consumption of energy-dense snacks – tax**

*Imagine the government is considering a new policy to increase the price of high calorie snacks by 10% to help people eat less.*

*Research shows that the introduction of this new policy will reduce the number of high calorie snacks people eat.*

**Group 3b: Achieving behaviour change – consumption of meat – tax**

*Imagine the government is considering a new policy to increase the price of meat products by 10% to help people eat less.*

*Research shows that the introduction of this new policy will reduce the number of meat products people eat.*

**Group 3c: Achieving behaviour change – consumption of alcohol – tax**

*Imagine the government is considering a new policy to increase the price of alcoholic drinks by 10% to help people drink less.*

*Research shows that the introduction of this new policy will reduce the number of alcoholic drinks people drink.*

**Group 4a: Behaviour change – consumption of energy-dense snacks – availability**

*Imagine the government is considering a new policy to decrease the number of high calorie snacks and increase the number of low calorie snacks in supermarkets.*

*Research shows that the introduction of this new policy will reduce the number of high calorie snacks people eat.*

**Group 4b Behaviour change – consumption of meat – availability**

*Imagine the government is considering a new policy to decrease the number of meat products and increase the number of plant-based foods in supermarkets.*

*Research shows that the introduction of this new policy will reduce the number of meat products people eat.*

**Group 4c: Behaviour change – consumption of alcohol – availability**

*Imagine the government is considering a new policy to decrease the number of alcoholic drinks and increase the number of alcohol-free drinks in supermarkets.*

*Research shows that the introduction of this new policy will reduce the number of alcoholic drinks people drink.*

**ONE BENEFIT**

**Group 5a: Achieving behaviour change + one benefit – consumption of energy-dense snacks – tax** (participants in this group were randomised to which benefit they read about)

*Imagine the government is considering a new policy to increase the price of high calorie snacks by 10% to help people eat less.*

*Research shows that the introduction of this new policy will reduce the number of high calorie snacks people eat. This will cut the number of people who get cancer.*

**OR**

*Imagine the government is considering a new policy to increase the price of high calorie snacks by 10% to help people eat less.*

*Research shows that the introduction of this new policy will reduce the number of high calorie snacks people eat. This will cut NHS costs.*

**OR**

*Imagine the government is considering a new policy to increase the price of high calorie snacks by 10% to help people eat less.*

*Research shows that the introduction of this new policy will reduce the number of high calorie snacks people eat. This will cut environmental harms.*

**Group 5b: Achieving behaviour change + one benefit – consumption of meat – tax** (participants in this group were randomised to which benefit they read about)

*Imagine the government is considering a new policy to increase the price of meat products by 10% to help people eat less.*

*Research shows that the introduction of this new policy will reduce the number of meat products people eat. This will cut the number of people who get cancer.*

**OR**

*Imagine the government is considering a new policy to increase the price of meat products by 10% to help people eat less.*

*Research shows that the introduction of this new policy will reduce the number of meat products people eat. This will cut NHS costs.*

**OR**

*Imagine the government is considering a new policy to increase the price of meat products by 10% to help people eat less.*

*Research shows that the introduction of this new policy will reduce the number of meat products people eat. This will cut environmental harms.*

**Group 5c: Achieving behaviour change + one benefit – consumption of alcohol – tax** (participants in this group were randomised to which benefit they read about)

*Imagine the government is considering a new policy to increase the price of alcoholic drinks by 10% to help people drink less.*

*Research shows that the introduction of this new policy will reduce the number of alcoholic drinks people drink. This will cut the number of people who get cancer.*

**OR**

*Imagine the government is considering a new policy to increase the price of alcoholic drinks by 10% to help people drink less.*

*Research shows that the introduction of this new policy will reduce the number of alcoholic drinks people drink. This will cut NHS costs.*

**OR**

*Imagine the government is considering a new policy to increase the price of alcoholic drinks by 10% to help people drink less.*

*Research shows that the introduction of this new policy will reduce the number of alcoholic drinks people drink. This will cut environmental harms*

**Group 6a: Achieving behaviour change + one benefit – consumption of energy-dense snacks – availability** (participants in this group were randomised to which benefit they read about)

*Imagine the government is considering a new policy to decrease the number of high calorie snacks and increase the number of low calorie snacks in supermarkets.*

*Research shows that the introduction of this new policy will reduce the number of high calorie snacks people eat. This will cut the number of people who get cancer.*

**OR**

*Imagine the government is considering a new policy to decrease the number of high calorie snacks and increase the number of low calorie snacks in supermarkets.*

*Research shows that the introduction of this new policy will reduce the number of high calorie snacks people eat. This will cut NHS costs.*

**OR**

*Imagine the government is considering a new policy to decrease the number of high calorie snacks and increase the number of low calorie snacks in supermarkets.*

*Research shows that the introduction of this new policy will reduce the number of high calorie snacks people eat. This will cut environmental harms*

**Group 6b: Achieving behaviour change + one benefit – consumption of meat – availability** (participants in this group were randomised to which benefit they read about)

*Imagine the government is considering a new policy to decrease the number of meat products and increase the number of plant-based foods in supermarkets.*

*Research shows that the introduction of this new policy will reduce the number of meat products people eat. This will cut the number of people who get cancer.*

**OR**

*Imagine the government is considering a new policy to decrease the number of meat products and increase the number of plant-based foods in supermarkets.*

*Research shows that the introduction of this new policy will reduce the number of meat products people eat. This will cut NHS costs.*

**OR**

*Imagine the government is considering a new policy to decrease the number of meat products and increase the number of plant-based foods in supermarkets.*

*Research shows that the introduction of this new policy will reduce the number of meat products people eat. This will cut environmental harms.*

**Group 6c: Achieving behaviour change + one benefit – consumption of alcohol – availability** (participants in this group were randomised to which benefit they read about)

*Imagine the government is considering a new policy to decrease the number of alcoholic drinks and increase the number of alcohol-free drinks in supermarkets.*

*Research shows that the introduction of this new policy will reduce the number of alcoholic drinks people drink. This will cut the number of people who get cancer.*

**OR**

*Imagine the government is considering a new policy to decrease the number of alcoholic drinks and increase the number of alcohol-free drinks in supermarkets.*

*Research shows that the introduction of this new policy will reduce the number of alcoholic drinks people drink. This will cut NHS costs.*

**OR**

*Imagine the government is considering a new policy to decrease the number of alcoholic drinks and increase the number of alcohol-free drinks in supermarkets.*

*Research shows that the introduction of this new policy will reduce the number of alcoholic drinks people drink. This will cut environmental harms.*

**THREE BENEFITS**

**Group 7a: Achieving behaviour change + three benefit – consumption of energy-dense snacks – tax**

*Imagine the government is considering a new policy to increase the price of high calorie snacks by 10% to help people eat less.*

*Research shows that the introduction of this new policy will reduce the number of high calorie snacks people eat. This will cut the following:*

- *The number of people who get cancer*
- *NHS costs*
- *Environmental harms*

**Group 7b: Achieving behaviour change + three benefit – consumption of meat – tax**

*Imagine the government is considering a new policy to increase the price of meat products by 10% to help people eat less.*

*Research shows that the introduction of this new policy will reduce the number of meat products people eat. This will cut the following:*

- *The number of people who get cancer*
- *NHS costs*
- *Environmental harms*

**Group 7c: Achieving behaviour change + three benefit – consumption of alcohol – tax**

*Imagine the government is considering a new policy to increase the price of alcoholic drinks by 10% to help people drink less.*

*Research shows that the introduction of this new policy will reduce the number of alcoholic drinks people drink. This will cut the following:*

- *The number of people who get cancer*
- *NHS costs*
- *Environmental harms*

**Group 8a: Achieving behaviour change + three benefit – consumption of energy-dense snacks – availability**

*Imagine the government is considering a new policy to decrease the number of high calorie snacks and increase the number of low calorie snacks in supermarkets*

*Research shows that the introduction of this new policy will reduce the number of high calorie snacks people eat. This will cut the following:*

- *The number of people who get cancer*
- *NHS costs*
- *Environmental harms*

**Group 8b: Achieving behaviour change + three benefit – consumption of meat – availability**

*Imagine the government is considering a new policy to decrease the number of meat products and increase the number of plant-based foods in supermarkets*

*Research shows that the introduction of this new policy will reduce the number of meat products people eat.  This will cut the following:*

- *The number of people who get cancer*
- *NHS costs*
- *Environmental harms*

**Group 8c: Achieving behaviour change + three benefit – consumption of alcohol – availability**

*Imagine the government is considering a new policy to decrease the number of alcoholic drinks and increase the number of alcohol-free drinks in supermarkets*

*Research shows that the introduction of this new policy will reduce the number of alcoholic drinks people drink. This will cut the following:*

- *The number of people who get cancer*
- *NHS costs*
- *Environmental harms*

**Measurement of Value**

1. Value assigned to different benefits (population health vs healthcare costs vs planetary health) assessed using:

- a ranking measure where participants were asked to choose the most important and second most important benefit to them
- a rating measure where participants were asked to rate on a 7-point scale how important each benefit was to them (For :

Ranking measures:

1. *Which of the following is the most important issue to you (tick one)?”*

- *Everybody in the population having good health, including not getting cancer*
- *Everything being done to protect the environment, including preventing climate change*
- *Everything being done to improve the quality of healthcare, including reducing unnecessary costs*

1. *“Which of the following is the second most important issue to you (tick one)”*

- *Everybody in the population having good health, including not getting cancer*
- *Everything being done to protect the environment, including preventing climate change*
- *Everything being done to improve the quality of healthcare, including reducing unnecessary costs*

Rating measure:

*Please rate how important the following are for you:*

- *Everybody in the population having good health, including not getting cancer (*1=not at all important; 7=very important)
- *Everything being done to protect the environment, including preventing climate change (*1=not at all important; 7=very important)
- *Everything being done to improve the quality of healthcare, including reducing unnecessary costs (*1=not at all important; 7=very important)

**Tables and figures**

**Table S1:** Number of participants randomised to each experimental group

|  | **Evidence of effectiveness** | | | | | | | | | | | |
| --- | --- | --- | --- | --- | --- | --- | --- | --- | --- | --- | --- | --- |
|  | No message | | | Effectiveness for changing behaviour | | | Effectiveness for changing behaviour  +one benefit | | | Effectiveness for changing behaviour  +three benefits | | |
| **Behaviour** | Tax | Availability | **Across policies** | Tax | Availability | **Across policies** | Tax | Availability | **Across policies** | Tax | Availability | **Across policies** |
| Energy-dense food consumption | n=194 | n= 189 | n=384 | n=194 | n=185 | n=379 | n=191 | n=202 | n=393 | n=194 | n=186 | n= 380 |
| Meat consumption | n=194 | n=189 | n=384 | n=190 | n=168 | n= 358 | n=181 | n=190 | n=371 | n=180 | n=196 | n= 376 |
| Alcohol consumption | n=197 | n=201 | n=398 | n=181 | n=196 | n= 377 | n=189 | n=213 | n=402 | n=215 | n=199 | n=414 |
| **Across behaviours** | n=585 | n=580 | n=1165 | n=565 | n= 549 | n= 1114 | n=560 | n= 606 | n=1166 | n=590 | n=581 | n=1171 |

**Table S2:** Demographic characteristics (weighted) according to group

|  | **Group** | | | | | | | | | | | |
| --- | --- | --- | --- | --- | --- | --- | --- | --- | --- | --- | --- | --- |
|  | **1a (n=194)** | **1b (n=194)** | **1c (n=197)** | **2a (n=189)** | **2b (n=189)** | **2c (n=201)** | **3a (n=194)** | **3b (n=190)** | **3c**  **(n=181)** | **4a (n=185)** | **4b (n=168)** | **4c**  **(n=196)** |
| **Age(years (sd)** | 47.4 (17.2) (median=47.8) | 48.6 (17.2) (median=51) | 46.5 (18.1)  (median=45) | 46.8 (15.1)  (median=48) | 50.0 (16.4)  (median=51) | 46.8 (15.7)  (median=47) | 46.3 (17.9)  (median=45) | 48.4 (16.3)  (median=48) | 52.5 (16.0)  (median=53.2) | 46.8 (16.9)  (median=44.8) | 48.9 (16.6)  (median=48.1) | 49.2 (16.3)  (median=49) |
| **BMI (kg/m^2^ (sd)**) | 26.9 (6.1) | 27.5 (5.9) | 26.4 (5.1) | 27.3 (5.7) | 27.3 (6.0) | 26.7 (5.7) | 26.6 (5.1) | 27.6 (5.4) | 26.6 (4.9) | 26.9 (5.7) | 27.3 (5.7) | 27.2 (5.8) |
| **Sex (n (%))**  Female  Male | 92 (47%)  102 (53%) | 104(54%)  89 (46%) | 95 (48%)  102(52%) | 88 (47%)  101 (53%) | 91 (48%)  98 (52%) | 104 (52%)  97 (48%) | 107 (55%)  87 (45%) | 96 (50%)  94 (50%) | 93 (51%)  88 (49%) | 85 (46%)  100 (54%) | 77 (46%)  90 (54%) | 84 (43%)  113 (57%) |
| E**ducation (n (%))**  Low  Medium  High  Prefer not to say | 63 (32%)  30 (16%)  85 (44%)  16 (8%) | 68 (35%)  27 (14%)  87 (45%)  12 (6%) | 66 (34%)  38 (19%)  79 (40%)  13 (7%) | 49 (26%)  38 (20%)  97 (51%)  6 (3%) | 69 (36%)  22 (12%  91 (48%)  7 (4%) | 56 (28%)  34 (17%)  110 (55%)  1 (0.5%) | 75 (39%)  47 (24%)  64 (33%)  8 (4%) | 58 (30%)  37 (29%)  86 (45%)  9 (5%) | 78 (43%)  28 (15%)  71 (39%)  4 (2%) | 48 (26%)  41 (22%)  86 (47%)  10 (5%) | 43 (26%)  32 (19%)  83 (10%)  9 (5%) | 68 (34%)  39 (20%)  84 (43%)  6 (3.%) |
| **SES**  **(n (%))**  Low  Medium  High | 55 (28%)  102 (52%)  38 (19%) | 59 (31%)  100 (51%)  35 (18%) | 50 (26%)  106 (54%)  40 (20%) | 60 (32%)  84 (44%)  46 (24%) | 55 (29%)  94 (50%)  40 (21%) | 61 (30%)  111 (55%)  29 (14%) | 47 (24%)  105 (54%)  42 (22%) | 60 (32%)  87 (46%)  43 (23%) | 45 (25%)  96 (53%)  39 (22%) | 53 (29%)  96 (52%)  35 (19%) | 52 (31%)  79 (47%)  36 (22%) | 41 (21%)  96 (49%)  59 (30%) |
| **Ethnicity (n (%))**  White  Mixed  Asian  Black  Other/  Prefer not to say | 181 (93%)  2 (1%)  5 (3%)  4 (2%)  3 (1%) | 172(90%)  4 (2%)  9 (5%)  2 (1%)  5 (3%) | 184(93%)  6 (3%)  4 (2%)  3 (2%)  0 (0%) | 179 (94%)  1 (1%)  4 (2%)  0 (0%)  6 (3%) | 179 (95%)  5 (3%)  3 (2%)  1 (0.5%)  1 (0.5%) | 187 (93.0%)  1 (1%)  7 (3%)  1 (1%)  5 (2%) | 186 (95%)  4 (2%)  2 (1%)  2 (1%)  1 (1%) | 182(96%)  2 (1%)  1 (1%)  5 (2%)  0 (0%) | 174 (97%)  1 (1%)  4 (2%)  0 (0%)  1 (1%) | 174 (94%)  3 (2%)  6 (3%)  0 (0%)  2 (3%) | 157(93%)  3 (2%)  4 (2%)  2 (1%)  2 (3%) | 188 (95%)  3 (1%)  1 (1%)  3 (2%)  2 (1%) |
| I**ncome**  **(n (%))**  Low  Medium  High  Prefer not to say | 59 (30%)  53 (27%)  31 (16%)  51 (26%) | 59 (31%)  51 (26%)  33 (17%)  50 (26%) | 52 (26%)  52 (26%)  33 (17%)  59 (30%) | 48 (25%)  56 (30%)  34 (18%)  50 (27%) | 55 (29%)  35 (19%)  45 (24%)  53 (28%) | 44 (22%)  62 (31%)  56 (28%)  39 (19%) | 59 (31%)  60 (31%)  33 (17%)  41 (21%) | 41 (22%)  56 (30%)  42 (22%)  49 (26%) | 46 (26%)  59 (33%)  28 (16%)  45 (25%) | 53 (29%)  67 (36%)  33 (18%)  30 (16%) | 48 (29%)  44 (26%)  43 (26%)  32 (19%) | 61 (32%)  54 (28%)  31 (16%)  46 (24%) |

|  | **5a (n=191)** | **5b (n=181)** | **5c**  **(n=189)** | **6a (n=202)** | **6b (n=190)** | **6c (n=213)** | **7a**  **(n=194)** | **7b**  **(n=180)** | **7c (n=215)** | **8a (n=186)** | **8b (n=196)** | **8c (n=199)** |
| --- | --- | --- | --- | --- | --- | --- | --- | --- | --- | --- | --- | --- |
| **Age**  **(years)** | 50.0 (17.5)  (median=49) | 51.5 (15.9)  (median=52) | 48.5 (17.2)  median=49) | 49.2 (16.4)  (median=51.4) | 46.9 (17.0)  (median=47) | 48.2 (17.7)  (median=45) | 48.2 (17.6)  (median=49) | 49.7 (17.1)  (median=49.7) | 45.7 (18.4)  (median=44) | 48.6 (17.4) (median=49.7) | 47.7 (16.9)  (median=46) | 51.3 (17.9) (median=53) |
| **BMI (kg/m^2^**) | 26.7 (5.8) | 27.2 (5.2) | 27.2 (5.9) | 27.4 (6.2) | 26.0 (5.2) | 27.3 (5.4) | 27.1 (5.8) | 27.3 (6.4) | 27.3 (5.5) | 27.9 (5.9) | 27.3 (7.8) | 26.7 (5.7) |
| **Sex (n (%))**  Female  Male | 82 (43%)  108 (57%) | 82 (46%)  98 (54%) | 81 (43%)  108(57%) | 96 (47%)  106 (53%) | 93 (48.9%)  97 (51.1%) | 106 (49.8%)  107 (50.2%) | 99 (50.8%)  96 (49.2%) | 84 (46.7%)  96 (53.3%) | 108 (50.2%)  107 (49.8%) | 97 (51.9%)  90 (48.1%) | 97 (49.5%)  99 (50.5%) | 91 (45.7%)  108 (54.3%) |
| E**ducation (n (%))**  Low  Medium  High  Prefer not to say | 67 (35%)  29 (15%)  82 (43%)  13 (7%) | 74 (41%)  32 (18%)  69 (38%)  7 (4%) | 56 (30%)  40 (21%)  85 (43%)  8 (4%) | 72 (36%)  46 (23%)  75 (37%)  8 (4%) | 67 (35%)  30 (16%)  86 (45%)  7 (4%) | 76 (35%)  51 (24%)  80(37%)  7(3%) | 59 (30%)  44 (23%)  82 (42%)  9 (5%) | 59 (33%)  34 (19%)  84 (47%)  3 (2%) | 66 (31%)  44 (20%)  87 (40%)  19 (9%) | 57 (31%)  40 (21%)  82 (44%)  8 (4%) | 61 (31%)  36(18%)  94 (48%)  6 (3%) | 80 (40%)  28 (14%)  85 (43%)  6 (3%) |
| **SES**  **(n (%))**  Low  Medium  High | 46 (24%)  93 (49%)  52 (27%) | 37 (20%)  100(55%)  44 (24%) | 48 (25%)  98 (52%)  43 (23%) | 67 (33%)  92 (45%)  43(21%) | 55 (29%)  84 (44%)  52 (27%) | 54 (25%)  108 (51%)  51 (24%) | 58 (30%)  94 (48%)  42 (22%) | 5  5 (30%)  88 (49%)  38 (21%) | 66 (31%)  111 (52%)  38 (18%) | 61 (33%)  85 (5%)  41 (22%) | 60 (30%)  96 (49%)  41 (21%) | 48 (24%)  106 (53%)  45 (23%) |
| **Ethnicity (n (%))**  White  Mixed  Asian  Black  Other/  Prefer not to say | 172 (90%)  4 (2%)  5 (3%)  2 (1%)  8 (4%) | 174(96%)  3 (2%)  2 (1%)  1 (1%)  1 (1%) | 180(95%)  1 (0.5%)  5 (3%)  1 (0.5%)  2 (1%) | 191 (95%)  5 (2.5%)  5 (2.5%)  0 (0%)  1 (0%) | 184(96%)  1 (0.5%)  4 (2%)  0 (0.0%)  2 (1%) | 208 (97%)  1 (0.5%)  4 (2%)  1 (0.5%)  0 (0%) | 176 (90%)  2 (1%)  10 (5%)  1 (0%)  6 (3%) | 167(92%)  5 (3%)  6 (3%)  1 (1%)  2 (1%) | 199 (93%)  3 (1%)  3 (1%)  2 (1%)  8 (4%) | 177 (95%)  2 (1%)  6 (3%)  0 (0%)  2 (1%) | 182(93%)  2 (1%)  7 (4%)  2 (1%)  2 (1%) | 188 (94%)  1 (0.5%)  7 (3.5%)  1 (0.5%)  3 (1.5%) |
| I**ncome**  **(n (%))**  Low  Medium  High  Prefer not to say | 50 (26%)  53 (28%)  33 (17%)  54 (28%) | 51 (28%)  62 (35%)  27 (15%)  39 (23%) | 52 (28%)  48 (26%)  36 (19%)  51 (27%) | 61 (30%)  44 (22%)  41 (20%)  56 (28%) | 45 (24%)  46 (24%)  31 (16%)  68 (36%) | 64 (30%)  56 (26%)  38 (18%)  55 (26%) | 49 (26%)  49 (26%)  46 (24%)  46 (24%) | 57 (32%)  48 (27%)  43 (24%)  32 (18%) | 62 (29%)  63 (29%)  31 (14%)  59 (27%) | 52 (28%)  44 (24%)  34 (18%)  56 (30%) | 52 (27%)  55 (28%)  49 (25%)  39 (20%) | 62 (31%)  58 (29%)  31 (16%)  47 (24%) |

Group 1a=No message, snacks, tax

Group 1b=No message, meat, tax

Group 1c=N message, alcohol, tax

Group 2a= No message, snacks, availability

Group 2b= No message, meat, availability

Group 2c= No message, alcohol, availability

Group 3a=Behaviour change, snacks tax

Group 3b =Behaviour change, meat, tax

Group 3c= Behaviour change, alcohol, tax

Group 4a+ Behaviour change, snacks, availability

Group 4b+ Behaviour change, meat, availability

Group 4c=Behaviour change, alcohol, availability

Group 5a=One benefit, snacks, tax

Group 5b= One benefit, meat, tax

Group 5c= One benefit, alcohol, tax

Group 6a= One benefit, snacks, availability

Group 6b=One benefit, meat, availability

Group 6c=One benefit, alcohol, availability

Group 7a= Three benefits, snacks, tax

Group 7b= Three benefits, meat, tax

Group 7c= Three benefits, alcohol, tax

Group 8a= Three benefits, snacks, availability

Group 8b= Three benefits, meat, availability

Group 8c= Three benefits, alcohol, availability

**Table S3**: Policy support (mean (sd)) for each policy by targeted behaviour according to level of evidence of effectiveness communication

|  | **Evidence of effectiveness** | | | | | | | | | | | |
| --- | --- | --- | --- | --- | --- | --- | --- | --- | --- | --- | --- | --- |
|  | No message | | | Effectiveness for changing behaviour | | | Effectiveness for changing behaviour  +one benefit | | | Effectiveness for changing behaviour  +three benefits | | |
| **Behaviour** | Tax | Availability | Across policies | Tax | Availability | Across policies | Tax | Availability | Across policies | Tax | Availability | Across policies |
| Energy-dense food consumption | 3.93 *(1.90)* | 4.68  *(1.73)* | 4.32 *(1.87)* | 4.07 *(3.74)* | 4.70  *(1.65)* | 4.43 *(1.75)* | 4.35 *(1.97)* | 4.93  *(1.52)* | 4.65 *(1.76)* | 4.67 *(1.72)* | 4.97  *(1.57)* | 4.84 *(1.65)* |
| Meat consumption | 3.10 *(1.77)* | 3.94  *(1.98)* | 3.52 *(1.91)* | 3.19 *(1.91)* | 3.62  *(1.87)* | 3.44 *(1.88)* | 3.38 *(1.72)* | 3.99  *(1.79)* | 3.69 *(1.78)* | 3.75 *(2.02)* | 4.27  *(1.83)* | 4.02 *(1.93)* |
| Alcohol consumption | 4.12 *(1.99)* | 4.00  *(1.67)* | 4.08 *(1.83)* | 3.98 *(1.97)* | 3.94  *(1.65)* | 3.94 *(1.81)* | 4.21 *(1.82)* | 4.16  *(1.42)* | 4.19 *(1.62)* | 4.37 *(1.88)* | 4.55  *(1.68)* | 4.49 *(1.77)* |
| Across behaviours | 3.75 *(1.93)* | 4.22  *(1.84)* | 3.98 *(1.91)* | 3.78 *(1.92)* | 4.10  *(1.78)* | 3.94 *(1.86)* | 3.98 (*1.87)* | 4.37  *(1.45)* | 4.18 *(1.77)* | 4.30 *(1.89)* | 4.60  *(1.71)* | 4.45 *(1.81)* |

**Table S4**: Perceived policy effectiveness (mean (sd)) according to group

|  | **Evidence of effectiveness** | | | | | | | | | | | |
| --- | --- | --- | --- | --- | --- | --- | --- | --- | --- | --- | --- | --- |
|  | No message | | | Effectiveness for changing behaviour | | | Effectiveness for changing behaviour  +one benefit | | | Effectiveness for changing behaviour  +three benefits | | |
| **Behaviour** | Tax | Availability | Across policies | Tax | Availability | Across policies | Tax | Availability | Across policies | Tax | Availability | Across policies |
| Energy-dense food consumption | 3.22 (1.51) | 3.94 (1.62) | 3.61 (1.60) | 3.73 (1.56) | 3.99 (1.51) | 3.88 (1.53) | 3.49 (1.60) | 4.15 (1.41) | 3.85 (1.54) | 3.66 (1.44) | 4.14 (1.42) | 3.91 (1.45) |
| Meat consumption | 3.22 (1.52) | 3.75 (1.65) | 3.49 (1.60) | 3.39 (1.47) | 3.85 (1.55) | 3.64 (1.53) | 3.37 (1.43) | 3.94 (1.38) | 3.66 (1.44) | 3.62 (1.56) | 3.95 (1.51) | 3.80 (1.55) |
| Alcohol consumption | 3.36 (1.62) | 3.16 (1.57) | 3.26 (1.59) | 3.29 (1.58) | 3.39 (1.52) | 3.32 (1.55) | 3.55 (1.49) | 3.46 (1.44) | 3.49 (1.46) | 3.52 (1.66) | 3.61 (1.46) | 3.58 (1.57) |
| Across behaviours | 3.27 (1.54) | 3.64 (1.65 | 3.45 (1.60) | 3.48 (1.54) | 3.75 (1.54) | 3.61 (1.55) | 3.46 (1.50) | 3.86 (1.45) | 3.66 (1.49) | 3.61 (1.56) | 3.91 (1.48) | 3.76 (1.53) |

**Table S5**: ANOVA results: perceived policy effectiveness according to effectiveness communication, policy type and target behaviour

| **Source** | **Type III Sum of Squares** | **df** | **Mean Square** | **F** | **P** | **Partial Eta Squared** |
| --- | --- | --- | --- | --- | --- | --- |
| Intercept | 68319.379 | 1 | 68319.379 | 29515.282 | .000 | .851 |
| Evidence of effectiveness communication | 63.497 | 3 | 21.166 | 9.144 | .000 | .005 |
| Policy type | 139.540 | 2 | 69.770 | 30.142 | .000 | .011 |
| Behaviour targeted | 147.701 | 1 | 147.701 | 63.810 | .000 | .012 |
| Evidence of effectiveness communication * Policy type | 8.800 | 6 | 1.467 | .634 | .703 | .001 |
| Evidence of effectiveness communication * Behaviour targeted | 3.856 | 3 | 1.285 | .555 | .645 | .000 |
| Policy type * Behaviour targeted | 80.888 | 2 | 40.444 | 17.473 | .000 | .007 |
| Evidence of effectiveness communication  * Policy type * Behaviour targeted | 27.469 | 6 | 4.578 | 1.978 | .065 | .002 |
| Error | 12001.782 | 5185 | 2.315 |  |  |  |
| a. R Squared = .038 (Adjusted R Squared = .033) |  |  |  |  |  |  |

Table S6: Mean (sd) importance score assigned to each benefit (n=4616)

| **Benefit** | I**mportance score** |
| --- | --- |
| Population Health | 5.95 (1.46) |
| Planetary Health | 5.57 (1.57) |
| Healthcare Costs | 5.81 (1.37) |

**Figure S1**: Violin plot depicting the mean (± 1 SD) public support rating by group


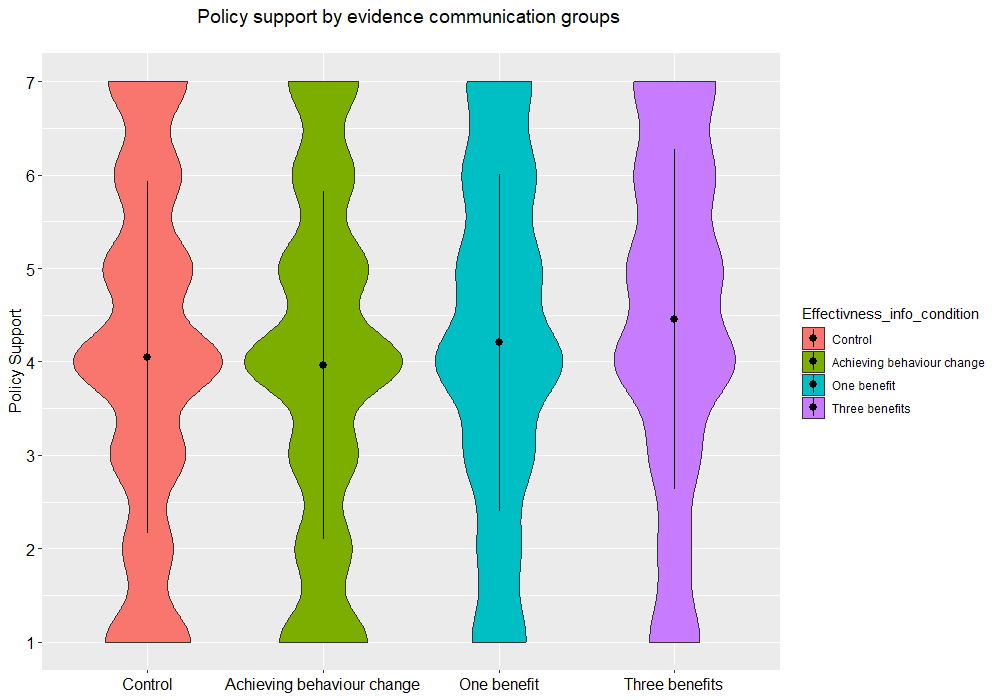


**Sensitivity analyses**

**Impact on Policy Support -Controlling for demographics and frequency of consumption**

| Source | **Type III Sum of Squares** | **df** | **Mean Square** | **F** | **P** | **Partial Eta Squared** |
| --- | --- | --- | --- | --- | --- | --- |
| Intercept | 2404.539 | 1 | 2404.539 | 787.467 | .000 | .133 |
| Frequency of consumption_meat products | 254.147 | 1 | 254.147 | 83.231 | .000 | .016 |
| Frequency of consumption_alcoholic products | 166.218 | 1 | 166.218 | 54.435 | .000 | .010 |
| Frequecy of consumption_energy dense snacks | .663 | 1 | .663 | .217 | .641 | .000 |
| Education | 79.537 | 1 | 79.537 | 26.048 | .000 | .005 |
| Ethnicity | .901 | 1 | .901 | .295 | .587 | .000 |
| Income | 4.260 | 1 | 4.260 | 1.395 | .238 | .000 |
| Age | 92.203 | 1 | 92.203 | 30.196 | .000 | .006 |
| Evidence of effectiveness communication | 212.916 | 3 | 70.972 | 23.243 | .000 | .013 |
| Policy type | 159.249 | 1 | 159.249 | 52.153 | .000 | .010 |
| Behaviour targeted | 703.740 | 2 | 351.870 | 115.235 | .000 | .043 |
| Evidence of effectiveness communication * Policy type | 6.930 | 3 | 2.310 | .756 | .518 | .000 |
| Evidence of effectiveness communication * Behaviour targeted | 10.019 | 6 | 1.670 | .547 | .773 | .001 |
| Policy type * Behaviour targeted | 92.963 | 2 | 46.481 | 15.222 | .000 | .006 |
| Evidence of effectiveness communication  * Policy type * Behaviour targeted | 29.293 | 6 | 4.882 | 1.599 | .143 | .002 |
| Error | 15707.250 | 5144 | 3.054 |  |  |  |
| a. R Squared = .108 (Adjusted R Squared = .102) |  |  |  |  |  |  |

**Impact on Policy Support - Using composite measure of policy support (controlling for demographics and frequency of consumption)**

| Source | Type III Sum of Squares | df | Mean Square | F | P | Partial Eta Squared |
| --- | --- | --- | --- | --- | --- | --- |
| Intercept | 2396.514 | 1 | 2396.514 | 826.212 | .000 | .138 |
| Frequency of consumption_meat products | 264.890 | 1 | 264.890 | 91.322 | .000 | .017 |
| Frequency of consumption_alcoholic products | 172.479 | 1 | 172.479 | 59.463 | .000 | .011 |
| Frequecy of consumption_energy dense snacks | .245 | 1 | .245 | .085 | .771 | .000 |
| Education | 75.802 | 1 | 75.802 | 26.133 | .000 | .005 |
| Ethnicity | .037 | 1 | .037 | .013 | .910 | .000 |
| Income | 5.183 | 1 | 5.183 | 1.787 | .181 | .000 |
| Age | 74.403 | 1 | 74.403 | 25.651 | .000 | .005 |
| Evidence of effectiveness communication | 196.619 | 3 | 65.540 | 22.595 | .000 | .013 |
| Policy type | 153.253 | 1 | 153.253 | 52.835 | .000 | .010 |
| Behaviour targeted | 690.380 | 2 | 345.190 | 119.006 | .000 | .044 |
| Evidence of effectiveness communication * Policy type | 6.607 | 3 | 2.202 | .759 | .517 | .000 |
| Evidence of effectiveness communication * Behaviour targeted | 8.547 | 6 | 1.424 | .491 | .815 | .001 |
| Policy type * Behaviour targeted | 90.567 | 2 | 45.284 | 15.612 | .000 | .006 |
| Evidence of effectiveness communication  * Policy type * Behaviour targeted | 35.134 | 6 | 5.856 | 2.019 | .060 | .002 |
| Error | 14920.707 | 5144 | 2.901 |  |  |  |
| a. R Squared = .111 (Adjusted R Squared = .106) |  |  |  |  |  |  |

**Impact on Policy Support - Using composite measure of policy support**

| Source | Type III Sum of Squares | df | Mean Square | F | P | Partial Eta Squared |
| --- | --- | --- | --- | --- | --- | --- |
| Intercept | 86981.332 | 1 | 86981.332 | 28684.924 | .000 | .847 |
| Evidence of effectiveness communication | 194.602 | 3 | 64.867 | 21.392 | .000 | .012 |
| Policy type | 175.198 | 1 | 175.198 | 57.777 | .000 | .011 |
| Behaviour targeted | 675.969 | 2 | 337.985 | 111.461 | .000 | .041 |
| Evidence of effectiveness communication * Policy type | 4.859 | 3 | 1.620 | .534 | .659 | .000 |
| Evidence of effectiveness communication * Behaviour targeted | 8.155 | 6 | 1.359 | .448 | .847 | .001 |
| Policy type * Behaviour targeted | 103.895 | 2 | 51.948 | 17.131 | .000 | .007 |
| Evidence of effectiveness communication  * Policy type * Behaviour targeted | 32.364 | 6 | 5.394 | 1.779 | .099 | .002 |
| Error | 15722.482 | 5185 | 3.032 |  |  |  |
| a. R Squared = .071 (Adjusted R Squared = .066) |  |  |  |  |  |  |

**Impact on Perceived Policy Effectiveness -Controlling for demographics and frequency of consumption**

| Source | Type III Sum of Squares | df | Mean Square | F | P |
| --- | --- | --- | --- | --- | --- |
| Intercept | 2031.690 | 1 | 2031.690 | 899.686 | .000 |
| Frequency of consumption_meat products | 122.889 | 1 | 122.889 | 54.419 | .000 |
| Frequency of consumption_alcoholic products | 63.608 | 1 | 63.608 | 28.167 | .000 |
| Frequecy of consumption_energy dense snacks | .002 | 1 | .002 | .001 | .974 |
| Ethnicity | 2.613 | 1 | 2.613 | 1.157 | .282 |
| Education | 23.522 | 1 | 23.522 | 10.416 | .001 |
| Age | 3.597 | 1 | 3.597 | 1.593 | .207 |
| Income | 37.077 | 1 | 37.077 | 16.419 | .000 |
| Evidence of effectiveness communication | 67.954 | 3 | 22.651 | 10.031 | .000 |
| Policy type | 133.589 | 1 | 133.589 | 59.157 | .000 |
| Behaviour targeted | 134.703 | 2 | 67.351 | 29.825 | .000 |
| Evidence of effectiveness communication * Policy type | 6.417 | 3 | 2.139 | .947 | .417 |
| Evidence of effectiveness communication * Behaviour targeted | 4.610 | 6 | .768 | .340 | .916 |
| Policy type * Behaviour targeted | 75.168 | 2 | 37.584 | 16.643 | .000 |
| Evidence of effectiveness communication  * Policy type * Behaviour targeted | 33.122 | 6 | 5.520 | 2.445 | .023 |
| Error | 11616.286 | 5144 | 2.258 |  |  |
| a. R Squared = .061 (Adjusted R Squared = .056) |  |  |  |  |  |

**Exploratory Moderation Analysis**

**Moderator: Value assigned to Population Health**

**Rating measure**

Model Summary

| R | R-sq | MSE | F | df1 | df2 | p |
| --- | --- | --- | --- | --- | --- | --- |
| .0905 | .0082 | 3.4585 | 4.2398 | 3.0000 | 1542.0000 | .0054 |

Model

|  | coeff | SE | t | P | LLCI | ULCI |
| --- | --- | --- | --- | --- | --- | --- |
| constant | 3.4363 | .2400 | 14.3167 | .0000 | 2.9655 | 3.9071 |
| Evidence of effectiveness communication | .2159 | .2385 | .9052 | .3655 | -.2520 | .6838 |
| Population Health Rating | .1023 | .0391 | 2.6159 | .0090 | .0256 | .1790 |
| Evidence of effectiveness communication *Population Health Rating | -.0162 | .0389 | -.4174 | .6765 | -.0924 | .0600 |

Test(s) of highest order unconditional interaction(s):

|  | R2-chng | F | df1 | df2 | p |
| --- | --- | --- | --- | --- | --- |
| Evidence of effectiveness communication *Population Health Rating | .0001 | .1742 | 1.0000 | 1542.0000 | .6765 |

**Ranking measure**

Model Summary

| R | R-sq | MSE | F | df1 | df2 | p |
| --- | --- | --- | --- | --- | --- | --- |
| .0806 | 0065 | 3.4643 | 3.3651 | 3.0000 | 1542.0000 | .0180 |

Model

|  | coeff | SE | t | P | LLCI | ULCI |
| --- | --- | --- | --- | --- | --- | --- |
| constant | 3.7769 | .1312 | 28.7873 | .0000 | 3.5195 | 4.0342 |
| Evidence of effectiveness communication | .2933 | .1288 | 2.2769 | .0229 | .0406 | .5461 |
| Population Health Rating | .1614 | .0711 | 2.2713 | .0233 | .0220 | .3008 |
| Evidence of effectiveness communication *Population Health Ranking | -.1035 | .0687 | -1.5070 | .1320 | -.2382 | .0312 |

Test(s) of highest order unconditional interaction(s):

|  | R2-chng | F | df1 | df2 | p |
| --- | --- | --- | --- | --- | --- |
| Evidence of effectiveness communication *Population Health Ranking | .0015 | 2.2709 | 1.0000 | 1542.0000 | .1320 |

**Moderator: Value assigned to Planetary health**

**Rating measure**

Model Summary

| R | R-sq | MSE | F | df1 | df2 | p |
| --- | --- | --- | --- | --- | --- | --- |
| .2046 | .0419 | 3.3835 | 22.2966 | 3.0000 | 1531.0000 | .0180 |

Model

|  | coeff | SE | t | P | LLCI | ULCI |
| --- | --- | --- | --- | --- | --- | --- |
| constant | 2.7667 | .2146 | 12.8946 | .0000 | 2.3458 | 3.1876 |
| Evidence of effectiveness communication | -.0481 | .2049 | -.2347 | .8145 | -.4500 | .3539 |
| Planetary Health Rating | .2244 | .0364 | 6.1699 | .0000 | . 1530 | .2957 |
| Evidence of effectiveness communication * Planetary Health Ranking | .0341 | .0353 | .9671 | .3336 | -.0351 | .1032 |

Test(s) of highest order unconditional interaction(s):

|  | R2-chng | F | df1 | df2 | p |
| --- | --- | --- | --- | --- | --- |
| Evidence of effectiveness communication * Planetary Health Ranking | .0006 | .9354 | 1.0000 | 1531.0000 | .3336 |

**Ranking measure**

Model Summary

| R | R-sq | MSE | F | df1 | df2 | p |
| --- | --- | --- | --- | --- | --- | --- |
| .1176 | .0138 | 3.4824 | 7.1576 | 3.0000 | 1531.0000 | .0001 |

Model

|  | coeff | SE | t | P | LLCI | ULCI |
| --- | --- | --- | --- | --- | --- | --- |
| constant | 3.6253 | .1662 | 21.8077 | .0000 | 3.2992 | 3.9514 |
| Evidence of effectiveness communication | .0594 | .1631 | -.3642 | .7158 | -.3793 | .2605 |
| Planetary Health Rating | .1978 | .0735 | 2.6918 | .0072 | .0537 | .3419 |
| Evidence of effectiveness communication * Planetary Health Rating | .0896 | .0727 | 1.2323 | .2180 | -.0530 | .2323 |

Test(s) of highest order unconditional interaction(s):

|  | R2-chng | F | df1 | df2 | p |
| --- | --- | --- | --- | --- | --- |
| Evidence of effectiveness communication * Planetary Health Rating | .0010 | 1.5186 | 1.0000 | 1531.0000 | .2180 |

**Moderator: Value assigned to Healthcare costs**

**Rating measure**

Model Summary

| R | R-sq | MSE | F | df1 | df2 | p |
| --- | --- | --- | --- | --- | --- | --- |
| .0651 | .0042 | 3.4176 | 2.1777 | 3.0000 | 1537.0000 | .0888 |

Model

|  | coeff | SE | t | P | LLCI | ULCI |
| --- | --- | --- | --- | --- | --- | --- |
| constant | 3.4656 | .2488 | 13.9286 | .0000 | 2.9776 | 3.9537 |
| Evidence of effectiveness communication | .1143 | .2338 | .4890 | .6249 | -.3442 | .5728 |
| Healthcare Costs Rating | .0989 | .0413 | 2.3972 | .0166 | .0180 | .1799 |
| Evidence of effectiveness communication * Healthcare Costs Rating | -.0205 | .0392 | -.5230 | .6010 | -.0974 | .0564 |

Test(s) of highest order unconditional interaction(s):

|  | R2-chng | F | df1 | df2 | p |
| --- | --- | --- | --- | --- | --- |
| Evidence of effectiveness communication * Planetary Health Rating | .0002 | .2735 | 1.0000 | 1537.0000 | .6010 |

**Ranking measure**

Model Summary

| R | R-sq | MSE | F | df1 | df2 | p |
| --- | --- | --- | --- | --- | --- | --- |
| .1205 | .0145 | 3.3823 | 7.5452 | 3.0000 | 1537.0000 | .0001 |

Model

|  | coeff | SE | t | P | LLCI | ULCI |
| --- | --- | --- | --- | --- | --- | --- |
| constant | 4.7028 | .1525 | 30.8297 | .0000 | 4.4036 | 5.0020 |
| Evidence of effectiveness communication | -.1821 | .1550 | -1.1745 | .2404 | -.4861 | .1220 |
| Healthcare Costs Ranking | -.2995 | .0652 | -4.5938 | .0000 | -.4274 | -.1716 |
| Evidence of effectiveness communication * Healthcare Costs Ranking | .0804 | .0654 | 1.2298 | .2190 | -.0478 | .2086 |

Test(s) of highest order unconditional interaction(s):

|  | R2-chng | F | df1 | df2 | p |
| --- | --- | --- | --- | --- | --- |
| Evidence of effectiveness communication * Planetary Health Ranking | .0010 | 1.5124 | 1.0000 | 1537.0000 | .2190 |

**Primary analysis model diagnostics (analysis of ANOVA residuals)**

| **Case Processing Summary** |  |  |  |  |  |  |
| --- | --- | --- | --- | --- | --- | --- |
|  | Cases |  |  |  |  |  |
|  | Valid | Missing | Total |  |  |  |
|  | N | Percent | N | Percent | N | Percent |
| Residual for Policy_Support_1 | 4563 | 98.8% | 53 | 1.2% | 4616 | 100.0% |

| **Descriptives** |  |  |  |
| --- | --- | --- | --- |
|  | Statistic | Std. Error |  |
| Residual for Policy_Support_1 | Mean | -.0193 | .02642 |
|  | 95% Confidence Interval for Mean | Lower Bound | -.0711 |
|  |  | Upper Bound | .0324 |
|  | 5% Trimmed Mean | -.0100 |  |
|  | Median | .0148 |  |
|  | Variance | 3.184 |  |
|  | Std. Deviation | 1.78443 |  |
|  | Minimum | -3.97 |  |
|  | Maximum | 3.87 |  |
|  | Range | 7.84 |  |
|  | Interquartile Range | 2.42 |  |
|  | Skewness | -.096 | .036 |
|  | Kurtosis | -.735 | .072 |

| **Tests of Normality** |  |  |  |
| --- | --- | --- | --- |
|  | Kolmogorov-Smirnov^a^ |  |  |
|  | Statistic | df | Sig. |
| Residual for Policy_Support_1 | .051 | 4563 | .000 |
| a. Lilliefors Significance Correction |  |  |  |


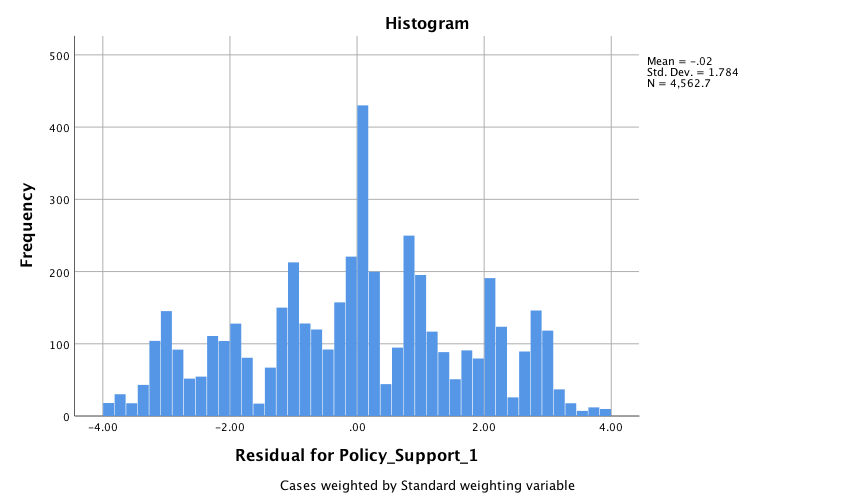


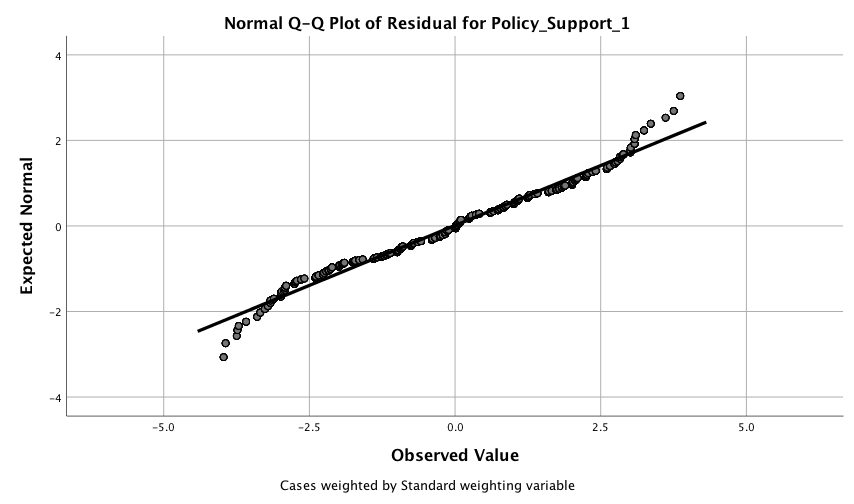


**Secondary analysis model diagnostics (analysis of ANOVA residuals)**

| **Descriptives** |  |  |  |
| --- | --- | --- | --- |
|  | Statistic | Std. Error |  |
| Residual for Perceived_effectiveness | Mean | -.0052 | .02250 |
|  | 95% Confidence Interval for Mean | Lower Bound | -.0493 |
|  |  | Upper Bound | .0389 |
|  | 5% Trimmed Mean | -.0229 |  |
|  | Median | .0440 |  |
|  | Variance | 2.310 |  |
|  | Std. Deviation | 1.52002 |  |
|  | Minimum | -3.17 |  |
|  | Maximum | 3.84 |  |
|  | Range | 7.01 |  |
|  | Interquartile Range | 2.24 |  |
|  | Skewness | .037 | .036 |
|  | Kurtosis | -.608 | .072 |

| **Tests of Normality** |  |  |  |
| --- | --- | --- | --- |
|  | Kolmogorov-Smirnov^a^ |  |  |
|  | Statistic | df | Sig. |
| Residual for Perceived_effectiveness | .048 | 4563 | .000 |
| a. Lilliefors Significance Correction |  |  |  |


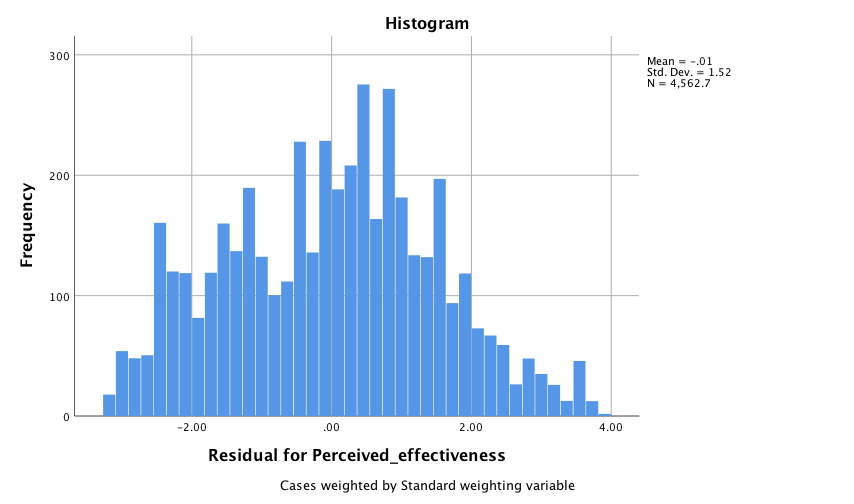


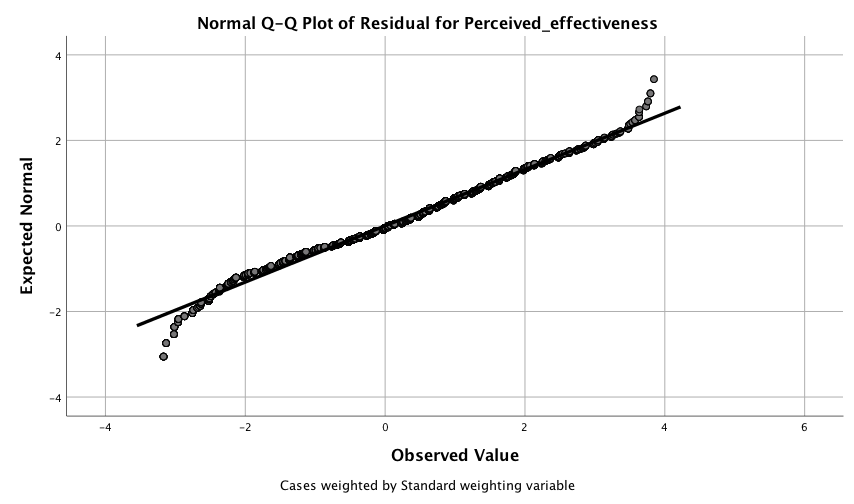


1. **Non-Reversal:** e.g. *This will cut the number of people who get cancer caused by eating too much*

   **Reversal**: e.g. *This will cut the number of people who get cancer caused by eating too much. If people ate and drank less, fewer people would have health problem*

   **Non-attribution**: e.g *This will cut the number of people who get caused by eating too much. The policy would help people eat less, meaning fewer people would have health problems* [↑](#footnote-ref-1)
2. **Carbon Emissions**: e.g. *This will cut carbon emissions caused by producing, processing and transporting food. If people ate less there would be fewer carbon emissions.*

   ***Environmental Harms:*** e.g. *This will cut carbon emissions caused by producing, processing and transporting food. If people ate less, there would be fewer harms to the environment.*

   **Both:** e.g. *This will cut carbon emissions caused by producing, processing and transporting food. If people ate less, there would be fewer carbon emissions and therefore fewer harms to the environment.*  [↑](#footnote-ref-2)
3. [↑](#footnote-ref-3)
